# A DNA Barcoding Method to Discriminate between the Model Plant *Brachypodium distachyon* and Its Close Relatives *B. stacei* and *B. hybridum* (Poaceae)

**DOI:** 10.1371/journal.pone.0051058

**Published:** 2012-12-11

**Authors:** Diana López-Alvarez, Maria Luisa López-Herranz, Alexander Betekhtin, Pilar Catalán

**Affiliations:** 1 Department of Agriculture and Environmental Sciences, University of Zaragoza, Huesca, Spain; 2 Department of Plant Anatomy and Cytology, University of Silesia, Katowice, Poland; University of Michigan, United States of America

## Abstract

**Background:**

*Brachypodium distachyon* s. l. has been widely investigated across the world as a model plant for temperate cereals and biofuel grasses. However, this annual plant shows three cytotypes that have been recently recognized as three independent species, the diploids *B. distachyon* (2n = 10) and *B. stacei* (2n = 20) and their derived allotetraploid *B. hybridum* (2n = 30).

**Methodology/Principal Findings:**

We propose a DNA barcoding approach that consists of a rapid, accurate and automatable species identification method using the standard DNA sequences of complementary plastid (*trn*LF) and nuclear (ITS, GI) loci. The highly homogenous but largely divergent *B. distachyon* and *B. stacei* diploids could be easily distinguished (100% identification success) using direct *trn*LF (2.4%), ITS (5.5%) or GI (3.8%) sequence divergence. By contrast, *B. hybridum* could only be unambiguously identified through the use of combined *trn*LF+ITS sequences (90% of identification success) or by cloned GI sequences (96.7%) that showed 5.4% (ITS) and 4% (GI) rate divergence between the two parental sequences found in the allopolyploid.

**Conclusion/Significance:**

Our data provide an unbiased and effective barcode to differentiate these three closely-related species from one another. This procedure overcomes the taxonomic uncertainty generated from methods based on morphology or flow cytometry identifications that have resulted in some misclassifications of the model plant and its allies. Our study also demonstrates that the allotetraploid *B. hybridum* has resulted from bi-directional crosses of *B. distachyon* and *B. stacei* plants acting either as maternal or paternal parents.

## Introduction

The impact of the new model plant *Brachypodium distachyon* on grass genomic research has gathered pace since the publication in 2010 of the full genome sequence of the diploid genotype Bd21 (2n = 10) by the International Brachypodium Initiative [1]. This taxon shows one of the smallest genome sizes of the monocots (272 Mb), together with a short life cycle (6 weeks), an inbreeding nature and a close relationship to the temperate cereals and forage crops [2]. These features make it an optimal model for the cultivated temperate cereals, wheats and barley, and other Poaceae. Over the last decade, more than 400 laboratories worldwide have worked on investigating the genomics, transcriptomics and metabolomics of *B. distachyon* [2], [3], [4]. Lines of research include studies on grain production, pathogen resistance, and tolerance to drought and to other abiotic stresses that could be transferred to cereal breeding programs [2], [3], [5], to those on cell wall analyses focused on the improvement of biofuel grass production [2], [5]. Other studies have highlighted the ecological plasticity of *B. distachyon* [6], [7], [8], adapted to different environmental conditions, as a suitable plant for ecosystem management and to prevent land erosion [7]. The compact genome of *B. distachyon*, which shows an extremely low amount of repetitive DNA [1], [2], has facilitated the construction of single-copy BAC libraries for comparative genomics and of derived mutagenized T-DNA and TILLING lines as a further aid to investigate gene expression effects under different natural and induced conditions in the model grass [2]. Additionally, large *B. distachyon* germplasm collections have been built at USDA (http://www.ars-grin.gov/npgs), and in several European and Mediterranean institutions [2], [3], [4], [9], [10], containing accessions with both economically and ecologically relevant traits and showing large phenetic and genotypic variation for on-going mapping projects.

The taxonomic and genomic identity of *B. distachyon* has been recently challenged by the evolutionary and systematic study of Catalán and coworkers [11]. Three cytotypes of *B. distachyon sensu lato* (s. l.) are known (2n = 10, 2n = 20 and 2n = 30) which were previously attributed to different ploidy levels of the same taxon *B. distachyon* s. l. (e. g., an autopolyploid series of individuals with x = 5 and 2n = 10 (2x), 20 (4x), 30 (6x) chromosomes; [12]). Catalan and coworkers demonstrated, through exhaustive phylogenetic, cytogenetic and phenotypic analyses, that the three cytotypes should in fact be treated as three different species: two diploids, each with a different chromosome base number, *B. distachyon* (x = 5, 2n = 10) and *B. stacei* (x = 10, 2n = 20), and their derived allotetraploid *B. hybridum* (x = 5+10, 2n = 30). *In-situ* GISH and rDNA and single-BAC FISH hybridizations, nucleolar dominance, and Comparative Chromosome Painting (CCP) analyses have conclusively demonstrated that the genomes of the two diploid species participated in the origin of the allopolyploid *B. hybridum* genome [11], [13], [14], [15], [16]. Genome size analyses provided further evidence that the genome size of *B. hybridum* (c. 1.265 pg/2C) resulted from the sum of the genomes of the two parental species [11]. Phylogenetic analyses of two plastid (*ndh*F, *trn*LF) and four nuclear (ITS, ETS, CAL, GI, DGAT) genes indicated that the more basally-diverged *B. stacei* and the more recently evolved *B. distachyon* emerged from two independent lineages, confirming their contribution as genome donors of *B. hybridum* [11]. Statistical analysis of morphometric traits showed that five characters (stomata leaf guard cell length, pollen grain length, upper glume length, lemma length, and awn length) significantly discriminated among the three species when they were grown under controlled greenhouse conditions [11]. However, although the three species can be differentiated through several phenotypic and cytogenetic traits, their direct identification is not always straightforward as wild populations show overlapping phenotypic variation for some characters and a similar diploid genome size (*B. distachyon* 0.631 pg/2C, *B. stacei* 0.564 pg/2C; [11], [17]). This has led to taxonomic uncertainty among, or even to taxonomic misclassifications of, the model species and its close allies when using currently employed identification methods such as morphology or flow cytometry (see Discussion).

The importance of *B. distachyon* and its recently split congeners, *B. stacei* and *B. hybridum*, has been underlined in newly addressed initiatives on re-sequencing 56 new accessions of *B. distachyon* and the *de-novo* genome sequencing of *B. stacei* and *B. hybridum*, a project undertaken by the Joint Genome Institute and the International *Brachypodium* Consortium (http://brachypodium.pw.usda.gov/files/resequencing_description_110822.pdf). The genomic features of the three species of this complex, which are characterized by similar, small genomes with low repetitive DNA content, make it an ideal group to investigate the mechanisms of polyploid hybrid speciation, paralleling those of the major cereal (*Triticum*) crops [2], [5]. The imminent genome sequences of *B. stacei* and *B. hybridum* will allow comparative genomic and functional genomic analyses on these diploid and polyploid grasses and their potential transfer to other cereals and forage crops. A large-scale phenomic study of a collection of different *B. distachyon* accessions, adapted to different selection pressures and currently undergoing re-sequencing (see above), is also under way (EPPN initiative; http://www.plant-phenotyping-network.eu/) and could be extended to *B. stacei* and *B. hybridum* (John Doonan, pers. comm). These analyses would be hindered, however, by the lack of a reliable method to differentiate the individuals of the three species. This is particularly problematic in natural admixed populations, where *B. hybridum* grows in sympatry with one or the other parental species [6], [11] López-Alvarez & Catalán, unpublished data]. Misidentified *B. stacei* and *B. hybridum* samples have also been found within the *B. distachyon* germplasm collections (see Discussion). Therefore, if the model plant is not one but three species, it is imperative to find an accurate and easily performed method to separate them. The DNA barcoding system offers a suitable approach to this problem.

From the several genes proposed as potential DNA barcodes for plants, the combination of the partial sequences of the plastid *rbc*L and *mat*K coding genes was selected as the preferred core sequence by the CBOL Plant Working Group [18]. These authors also recommended the use of other fragments in combination with the *rbc*L+*mat*K core to increase resolution within complex taxonomic groups. However, recent studies have proposed other, more variable genes as suitable candidates for the DNA barcoding of closely related plants [19], [20], [21]. Among these, the plastid *trn*LF region [20], [21], [22] and the nuclear rDNA ITS region [20], [23] have demonstrated their utility to discriminate different angiosperms at the species level in many groups, though they are not effective in all cases [21], [22], [24], [25]. A mini-barcoding fragment within the *trn*LF region, the P6 loop, has provided useful barcoding species-specific markers in ecological and dietary studies [22], [25]. Analyses of large angiosperm data sets have demonstrated, however, that the inclusion of the nuclear ITS region significantly increased the discriminatory power of the barcoding method beyond that based on the plastid molecules alone [23]. Despite the drawbacks posed by the multicopy ITS region in plants, such as the potential presence of paralogous and recombinant copies, and its predominant concerted evolution towards one of the parental ribotypes in the hybrid species [26], there is overall agreement on the value of its use as a barcoding tool for plants [20], [23]. In contrast, little consensus has been reached on the use of nuclear single-copy genes as barcoding molecules for plants. The problem stems from the inherent difficulty of finding appropriate unlinked and non-duplicated orthologous genes across a wide spectrum of angiosperms, capable of high-resolution species discrimination [20], [27]. Initial progress, however, has been put forward in some plant groups, where the selection of various taxonomically widespread single-copy orthologous genes (COS) has helped to diagnose species [28], [29], [30].

The complexity of the appropriate barcoding method is undoubtedly related to the complexity and nature of the group under study. Thus, taxonomically complex groups where species boundaries are narrowly defined [31], recently radiated species which show incomplete lineage sorting and/or few private mutations [21], and polyploids of hybrid origin (allopolyploids) that inherited a maternal plastid genome but a biparental nuclear genome are among the most problematic plants to be barcoded [20]. The *B. distachyon* – *B. stacei* – *B. hybridum* complex fits these characteristics. However, the short generation time of these annuals likely allowed the accumulation of a high number of mutations in their plastid and nuclear genomes. This probably resulted in significantly higher evolutionary rates among these species than those detected in perennial *Brachypodium* species [11]. Although Catalán and co-workers conducted phylogenetic analyses using a restricted sampling of representatives of *B. distachyon*, *B. stacei* and *B. hybridum* (including type materials of the three species), they found evidence of low intraspecific variation and of high interspecific divergence in the studied plastid and nuclear DNA sequences of the diploids *B. stacei* and *B. distachyon*. Regarding the allotetraploid *B. hybridum*, the evolutionary analyses indicated that this species apparently inherited its maternal cpDNA genome from *B. stacei*, the paternal nrDNA ribotypes from *B. distachyon*, and one copy each of the nDNA single-copy CAL, GI, and DGAT genes from both parents [11]. These findings suggested that the studied fragments could be used as barcodes to discriminate among the three related species.

The first major aim of this study was to test whether two genes that have been previously proposed as barcoding tools for different angiosperms, the plastid *trn*LF region and the nuclear ITS region (both included in the study of Catalán and co-workers [11]), could be used as barcodes to discriminate the model plant *B. distachyon* and its close relatives *B. stacei* and *B. hybridum* when a large sample of representatives of the three taxa was surveyed. Secondly, we wanted to test whether the use of the two molecules would suffice to identify *B. hybridum* or if a third nuclear single-copy gene is necessary to unambiguously characterize the allotetraploid. A third goal of our study was to investigate whether *B. stacei* and *B. distachyon* were, respectively, the maternal and paternal genome donors of all the studied *B. hybridum*, in order to test whether this species had a monophyletic or polyphyletic origin.

## Results

**Figure 1:**
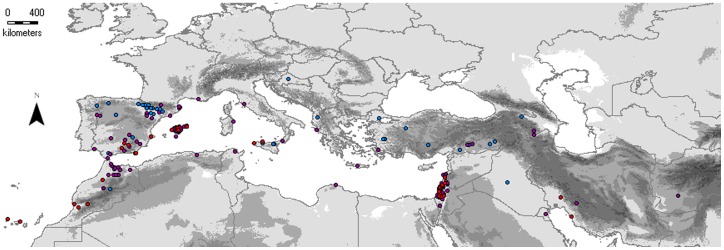
Geographical distribution of the studied taxa of the *Brachypodium distachyon* s. l. complex in their native circumMediterranean region. Blue, red and purple dots map, respectively, the localities of origin of the *B. distachyon*, *B. stacei* and *B. hybridum* samples.

Almost all the studied *B. distachyon*, *B. stacei* and *B. hybridum* samples (Fig. 1) were successfully amplified and sequenced for *trn*LF (n = 208; 93%), ITS (n = 210; 97%) and GI (n = 57; 98%) (Tables 1, 2). The total number of sequences obtained for each locus varied, ranging from 204 single-individual sequences for *trn*LF to 281 single-individual plus cloned sequences for ITS. In total, 342 single-individual plus cloned sequences were obtained for GI. All the new sequences have been deposited in Genbank under accession numbers JX665833-JX665848, JX665854-JX665898, JX665906-JX665998, JX666000-JX666038 (*trn*LF), JX665532-JX665546, JX665548-JX665550, JX665553-JX66557, JX66559-JX665618, JX66520-JX665623, JX665625-JX665627, JX665630-JX665638, JX665640-JX665761, JX665763-JX665832 (ITS) and JX666039- JX666041, JX666043-JX666095, JX666098-JX666241, JX967124-JX967262 (GI) (Table S1). A small number of incomplete or ambiguous sequences (4 *trn*LF, 20 ITS) were excluded from the haplotype network analysis but were used in the phylogenetic analyses (see Results below).

**Table 1:** Sources of *B. distachyon* (2n = 10), *B. stacei* (2n = 20) and *B. hybridum* (2n = 30) samples analysed for the *trn*LF, ITS and GI loci.

Taxa	Code	Locality	Chromosomes	Ploidy	*trn*LF		ITS		GI	
					Bdis	Bsta	Bdis	Bsta	Bdis	Bsta
*B.distachyon*	Bdis1	Spain: Huesca, Candasnos. PC&LM Bdis01	2n = 10	2x	h1		h1			
*B.distachyon*	Bdis2	Spain: Zaragoza, Castejón de Monegros. PC&LM Bdis04	2n = 10	2x	h2		h2			
*B.distachyon*	Bdis3	Spain: Huesca, Yaso. PC&LM Bdis08	2n = 10	2x	h2		h2			
*B.distachyon*	Bdis4	Spain: Huesca, Bierge. PC&LM Bdis09	2n = 10	2x	h2		h3			
*B.distachyon*	Bdis5	Spain: Huesca, Alquezar. PC&LM Bdis11	2n = 10	2x	h2		h1			
*B.distachyon*	Bdis6	Spain: Huesca, Jaca, Banaguas. PC&LM Bdis12	2n = 10	2x	h3		h2		h1	
*B.distachyon*	Bdis7	Spain: Huesca, Benabarre. PC&LM Bdis17	2n = 10	2x	h2		h2			
*B.distachyon*	Bdis8	Spain: Huesca, Arens. PC&LM Bdis18	2n = 10	2x	h2		h2			
*B.distachyon*	Bdis9	Spain: Huesca, Abizanda. PC&LM Bdis21	2n = 10	2x	h2		h4			
*B.distachyon*	Bdis10	Spain: Zaragoza, Miramont. PC&LM Bdis23	2n = 10	2x	h2		n.a			
*B.distachyon*	Bdis11	Spain: Navarra, Foz de Lumbier. PC&LM Bdis25	2n = 10	2x	h2		h1			
*B.distachyon*	Bdis12	Spain: Navarra, Puerto del Perdon. PC&LM Bdis27	2n = 10	2x	h3		h2			
*B.distachyon*	Bdis13	Spain: Lleida, Fondedou. PC&LM Bdis30	2n = 10	2x	h2		h2			
*B.distachyon*	Bdis14	Spain: Lleida, Castillo de Mur. PC&LM Bdis31	2n = 10	2x	h3		h2		h1	
*B.distachyon*	Bdis15	Spain: Lleida, Les Pallagues. PC&LM Bdis32	2n = 10	2x	h2		h1			
*B.distachyon*	Bdis16	Spain: Zaragoza, Belchite. PC&LM Bdis42	2n = 10	2x		h20	h1			
*B.distachyon*	Bdis17	Iraq: Salah ad Din, 4 km from Salahuddin USDA Bd21, type	2n = 10	2x	h2		h1		h1	
*B.distachyon*	Bdis18	Turkey: Kiresehir, Kaman. ABR1	2n = 10	2x	n.a		h5,h6 c		h1	
*B.distachyon*	Bdis19	France: Herault, Octon. ABR2 (Bdis306)	2n = 10	2x	h4		h2			
*B.distachyon*	Bdis20	Slovenia: Lubjana. ABR9 (Bdis384)	2n = 10	2x	h5		n.a			
*B.distachyon*	Bdis21	Spain: Huesca, Ibieca, Foces. PC&LM Bdis400	2n = 10	2x	h4		h2,h7 c			
*B.distachyon*	Bdis22	Spain: Huesca, Jaca, Guasillo. PC&LM Bdis401	2n = 10	2x	h4		h2 c			
*B.distachyon*	Bdis23	Spain: Albacete, Alcaraz. CS: Bd115F	2n = 10	2x	h6		h1, h8 c		h1,h2 c	
*B.distachyon*	Bdis24	Spain: León, Campohermoso. AM Leon	2n = 10	2x	h2		h2			
*B.distachyon*	Bdis25	Spain: Ourense, Sobrado. AM Sobr	2n = 10	2x	h2		h2			
*B.distachyon*	Bdis26	Spain: Valladolid, Íscar. AM Iscar	2n = 10	2x	h2		h2			
*B.distachyon*	Bdis27	Spain: Cádiz,Grazalema. AM Graz	2n = 10	2x	h7		h9		h1,h3 c	
*B.distachyon*	Bdis28	Turkey: Manisa. HB & JV BdTR1A	2n = 10*	2x	h8		h1			
*B.distachyon*	Bdis29	Turkey: Eskisehir. HB & JV BdTR2A	2n = 10*	2x	h4		h1			
*B.distachyon*	Bdis30	Turkey: Konya. HB & JV BdTR3A	2n = 10	2x	h9		h10			
*B.distachyon*	Bdis31	Turkey: Yozgat. HB & JV BdTR7B	2n = 10	2x	h4		n.a			
*B.distachyon*	Bdis32	Turkey: Bismil. MT & JV Bis-1	2n = 10*	2x	h9		n.a			
*B.distachyon*	Bdis33	Turkey: Gaziantep. MT & JV Gaz-2	2n = 10	2x	h4		n.a		h1	
*B.distachyon*	Bdis34	Turkey: Kahta. MT & JV Kah-1	2n = 10*	2x	h4		n.a			
*B.distachyon*	Bdis35	Turkey: Kozluk. MT & JV Koz-3	2n = 10*	2x	h10		n.a			
*B.distachyon*	Bdis36	Turkey: Tekirdag. MT & JV Tek (Tek1, Tek2, Tek8)	2n = 10*	2x	h11		h11, h12, h13 c			
*B.distachyon*	Bdis37	Turkey: Manisa. HB & JV BdTR1E	2n = 10*	2x	h4		n.a		h1,h7,h8,h9,h10 c	
*B.distachyon*	Bdis38	Israel: Julis. AB&SE BRA65	2n = 10*	2x	h12		h9		h11	
*B.distachyon*	Bdis39	Albania: 5 km on road from Elbasan to Tirana. AC Al1A	2n = 10	2x	h5		h14		h1	
*B.distachyon*	Bdis40	Albania: 5 km on road from Elbasan to Tirana. AC Al1B	2n = 10	2x	h5		h14			
*B.distachyon*	Bdis41	Albania: 5 km on road from Elbasan to Tirana. AC Al1C	2n = 10	2x	h5		h14			
*B.distachyon*	Bdis42	Albania: 5 km on road from Elbasan to Tirana. AC Al2A	2n = 10	2x	h5		h14			
*B.distachyon*	Bdis43	Albania: 5 km on road from Elbasan to Tirana. AC Al2B	2n = 10	2x	h5		h14			
*B.distachyon*	Bdis44	Armenia: Sjunik district; gorge of Vorotan rive. AC Arm2B	2n = 10	2x	h5		h1		h1	
*B.distachyon*	Bdis45	Armenia: Tavush district, Archi. AC Arm3G	2n = 10	2x	h2		h1			
*B.distachyon*	Bdis46	Armenia: Tavush district, Archi. AC Arm3I	2n = 10	2x	h5		h1			
*B.distachyon*	Bdis47	Italy: Sicily, Schilizzi. SH SLZ2	2n = 10	2x	h5		h13			
*B.distachyon*	Bdis48	Italy: Sicily, Lago di Piana degli Albanesi. SH LPA9	2n = 10	2x	h5		h3		h1	
*B.distachyon*	Bdis49	Italy: Sicily, Bronte. SH BNT2	2n = 10	2x	h5		h13			
*B.distachyon*	Bdis50	Italy: Sicily, Cesaro. SH CSR2	2n = 10	2x	h13		h3			
*B.distachyon*	Bdis51	Lebanon: Mohafaza Batroun Caza, Hannoush. RH-JA 25			h14		h9			
*B.distachyon*	Bdis52	Morocco: Al-Hoceina. 10 km of Ketama. RH-JA 95			h15		h15			
*B.distachyon*	Bdis53	Morocco: Rif Central 17 km from Ketama. RH-JA 97			h16		h15			
*B.distachyon*	Bdis54	Morocco: 14 km from Azrou. RH-JA 177			h17		n.a			
*B.distachyon*	Bdis55	Spain: Menorca, Ciutadella, Altruxt. DL&PC Bdis941*			h13		h16		h1	
*B.distachyon*	Bdis56	Spain: Mallorca, San Servera, ŃAmer. DL&PC Bdis1151*	2n = 10*	2x	h6		h17			
*B.stacei*	Bsta1	Spain: Formentera, Torrent, ABR114, type	2n = 20	2x		h20		h18, h19 c		h15
*B.stacei*	Bsta2	Israel: Hawalid. AB&SE BRA158	2n = 20*	2x		h20		h20		
*B.stacei*	Bsta3	Spain: Jaen, Baeza. CS Bd114F	2n = 20	2x		h20		h21 c		h16 c
*B.stacei*	Bsta4	Spain: Granada, Moclín. CS Bd129F	2n = 20	2x		h20		h22 c		h16,h17,h18 c
*B.stacei*	Bsta5	Spain: Alicante, Cabo de La Nao. CS Bd483F	2n = 20	2x		h21		h23, h24 c		
*B.stacei*	Bsta6	Iran: Fars, Kazeroon, Davan. MRR B100-H141				h20		h20		
*B.stacei*	Bsta7	Iran: 25 Km to Ramhorm from Ahwaz. MRR BdisIran2				h20		h20,h25,h26 c		
*B.stacei*	Bsta8	Iran: Asalooyeh to Booshehr MRR BdisIran1				h20				
*B.stacei*	Bsta9	Spain: Mallorca, La Victoria. INRA 13498BGVA				h20		h22		h19
*B.stacei*	Bsta10	Spain: Almería,Cabo de Gata. AM CG	2n = 20	2x		h20		h24		
*B.stacei*	Bsta11	Israel: Hazazon. AB&SE BRA1	2n = 20*	2x		h20		h20		
*B.stacei*	Bsta12	Israel: Amazyia. AB&SE BRA52	2n = 20*	2x		h20		h20		
*B.stacei*	Bsta13	Israel: Ziff junction. AB&SE BRA89	2n = 20*	2x		h20		h20		
*B.stacei*	Bsta14	Israel: Netanya. AB&SE BRA102	2n = 20*	2x		h20		h20		
*B.stacei*	Bsta15	Israel: Lakhish. AB&SE BRA135	2n = 20*	2x		h20		h20		
*B.stacei*	Bsta16	Israel: Bat Shelomo. AB&SE BRA191	2n = 20*	2x		h20		h20		
*B.stacei*	Bsta17	Israel: Modi’in. AB&SE BRA223	2n = 20*	2x		h20		h20		
*B.stacei*	Bsta18	Israel: Horvat Alon. AB&SE BRA252	2n = 20*	2x		h20		h20		
*B.stacei*	Bsta19	Israel: Zikhron Ya’akov. AB&SE BRA256	2n = 20*	2x		h20		h20		
*B.stacei*	Bsta20	Israel: Gilon-Zurit. AB&SE BRA271	2n = 20*	2x		h20		h20		h20
*B.stacei*	Bsta21	Israel: Yiftah. AB&SE BRA286	2n = 20*	2x		h20				
*B.stacei*	Bsta22	Spain: Almería, Cabo de Gata. EB: AL1	2n = 20	2x		h20				h15
*B.stacei*	Bsta23	Spain: Almería, Cabo de Gata. EB: AL5	2n = 20	2x						
*B.stacei*	Bsta24	Spain: Gomera, Agulo. INIA-CRF: NC050363	2n = 20	2x		h20		h27,h28,h29,h30 c		
*B.stacei*	Bsta25	Spain: Lanzarote, Teguise. INIA-CRF:NC050440	2n = 20	2x		h20		h18 c		
*B.stacei*	Bsta26	Italy: Sicily, Quattro Vanelle. SH QVL1	2n = 20	2x		h20		h31 c		h21
*B.stacei*	Bsta27	Italy: Sicily, Valdesi/Mondello. SH VMO1	2n = 20	2x		h20		h18		h22
*B.stacei*	Bsta28	Lebanon:Mohafaza, Caza Aley, Damour. RH-JA 27				h20		h18		
*B.stacei*	Bsta29	Palestine: Ramat-Gan near Tel-Aviv. RH-JA 36				n.a				
*B.stacei*	Bsta30	Israel: Dead Sea Valley: Mizpe Dragot. RH-JA 40				h22		h20		
*B.stacei*	Bsta31	Morocco: Agadir, Imouzzer Valley. RH-JA 58	2n = 20	2x		n.a		h18		
*B.stacei*	Bsta32	Morocco:Taliouine. RH-JA 62				h23		h22		
*B.stacei*	Bsta33	Morocco: Er Rachidia. RH-JA 179	2n = 20	2x		h20		h22		
*B.stacei*	Bsta34	Morocco: Agadir coast. RH-JA 180				n.a		h18		
*B.stacei*	Bsta35	Morocco: Essaouira. RH-JA 182				h20		h20		
*B.stacei*	Bsta36	Spain: Mallorca, Alcudia, c. Punta Negra. DL&PC Bdis621*	2n = 20	2x		h20		h22		h15
*B.stacei*	Bsta37	Spain: Mallorca, Campanet, Coves. DL&PC Bdis771*				h20		h22		
*B.stacei*	Bsta38	Spain: Mallorca, Sa Dragonera, Gambes. DL&PC Bdis891*				h21		h24		h15
*B.stacei*	Bsta39	Spain: Menorca, Es Mercadal, Toro. DL&PC Bdis951*	2n = 20	2x		h20	n.a			
*B.stacei*	Bsta40	Spain: Mallorca, Felenitx, San Salvador. DL&PC Bdis1091*	2n = 20	2x		h20				
*B.stacei*	Bsta41	Spain: Mallorca, Petra, Bonany. DL&PC Bdis1171*				h20		h32		
*B.stacei*	Bsta42	Spain: Jaen, Sierra de Quesada. Tiscar. JD:18_1	2n = 20	2x		h20		h22		h16
*B.stacei*	Bsta43	Spain: Jaen, Sierra de Quesada. Tiscar. JD:18_6	2n = 20	2x		h20		h22		
*B.hybridum*	Bhyb1	Spain: Zaragoza, La Alfranca 1. PC&LM :Bdis01	2n = 30	4x		h20	h33		h1,h5,h6 c	h4
*B.hybridum*	Bhyb2	Spain: Lleida, Menarguens. PC&LM:Bdis29	2n = 30*	4x		h20	h2		h1,h23,h24,h25,h26,h27, h105–h135c	
*B.hybridum*	Bhyb3	Spain: Girona,Roses, Castell de Trinitat. PC&LM:Bdis33	2n = 30*	4x		h20	n.a			
*B.hybridum*	Bhyb4	Spain: Girona, Cadaces, Port Lligat. PC&LM:Bdis34	2n = 30*	4x		h20	h3			
*B.hybridum*	Bhyb5	Spain: Girona, Cap de Lladró. PC&LM:Bdis36	2n = 30	4x		h20	h3			
*B.hybridum*	Bhyb6	Spain: Barcelona, El Prat Del Llobregat. PC&LM:Bdis38	2n = 30	4x						
*B.hybridum*	Bhyb7	Spain: Tarragona, Poble Nou del Delta. PC&LM:Bdis40	2n = 30*	4x		h20	n.a		h1,h29,h31 c	h28,h30 c
*B.hybridum*	Bhyb8	Spain: Teruel, Calaceite. PC&LM:Bdis41				h20	h3,h34,h35 c	h35	h1 c	h15,h32 c
*B.hybridum*	Bhyb9	France: Corsica, Bonifacio. ABR112 (Bdis383)	2n = 30	4x		h20	h3 c	h18 c		
*B.hybridum*	Bhyb10	Portugal: Lisboa 40. ABR Bdis385	2n = 30	4x		h20	h13,h37 c	h36 c	h34,h35,h36 c	h33,h37 c
*B.hybridum*	Bhyb11	Portugal: Lisboa, ABR113. type	2n = 30	4x		h20	h13,h38,h39 c		h1 c	h15 c
*B.hybridum*	Bhyb12	France: Aude. ABR110	2n = 30	4x		h20	h14			
*B.hybridum*	Bhyb13	Afghanistan: USDA PI219965, ABR117	2n = 30	4x		h20	h3 c		h40,h41,h42,h43, h44c	h38,h39 c
*B.hybridum*	Bhyb14	Spain: Zaragoza, La Alfranca. PC&LM:Bdis402	2n = 30	4x		h24	h2 c	h18 c		
*B.hybridum*	Bhyb15	Spain: Girona, Cadaques. PC&LM:Bdis403	2n = 30	4x		h20	h13 c	h18 c		
*B.hybridum*	Bhyb16	Italy: Campania, Vilammare. MA JC5338				h25				
*B.hybridum*	Bhyb17	Iran: Kohkiloyeh, Yasooj. MRR:B98-H137				h20				
*B.hybridum*	Bhyb18	Iran: Kohkiloyeh, Gchaemich T.Chogan. MRR B99-H138				h20	h41,h42 c	h20,h40,h42 c	h47 c	h15,h45,h46 c
*B.hybridum*	Bhyb19	Crete: INRA Cre0				h20	h13 c	h18,h43 c	h1 c	h48 c
*B.hybridum*	Bhyb20	Spain: Cordoba, Guadalcazar. INRA Esp1				h20	h2			
*B.hybridum*	Bhyb21	France: Esterel, Puy du Pertus. INRA Est0				h26				
*B.hybridum*	Bhyb22	Spain: Almeria, Cabo de Gata. ST SEV268823				h20	h1 c	h18 c		
*B.hybridum*	Bhyb23	Italy: Pian di Rocca, Grosetto. Orto Botanico 91_142	2n = 30*	4x		h20	h44 c	h18 c		
*B.hybridum*	Bhyb24	Spain: Ciudad Real,Ruidera. AM Rui	2n = 30	4x		h20	h3			
*B.hybridum*	Bhyb25	Spain: Córdoba,Los Pedroches. AM Copre	2n = 30	4x		h20	h3			
*B.hybridum*	Bhyb26	Spain: Jaen,La Cimbarra. AM Jcim	2n = 30	4x	h18		h45		h50 c	h26,h49,h51 c
*B.hybridum*	Bhyb27	Spain: Murcia,Moratalla. AM Mmor	2n = 30	4x		h20	h2			
*B.hybridum*	Bhyb28	Spain: Murcia,Espuña. AM Espuña	2n = 30	4x		h20		h18	h1,h52 c	h15,h53,h54 c
*B.hybridum*	Bhyb29	Spain: Granada,Baza. AM Gbaz	2n = 30	4x		h20	h1			
*B.hybridum*	Bhyb30	Spain: Huelva,Lepe. AM Lepe	2n = 30	4x	h19		h45		h55,h56,h58 c	h57 c
*B.hybridum*	Bhyb31	Spain: Granada,Nigüelas. AM Durcal	2n = 30	4x		h20	h46			
*B.hybridum*	Bhyb32	Spain: Málaga,Ronda. AM Mronda	2n = 30	4x		h20	h3			
*B.hybridum*	Bhyb33	Spain: Cádiz,Algeciras. AM Calge	2n = 30	4x		h20	h3			
*B.hybridum*	Bhyb34	Portugal: Tras-os-Montes,Mogadouro. AM Mog	2n = 30	4x		h20	h3		h1c, h142–h143 c	h15,h59,h144–h149 c
*B.hybridum*	Bhyb35	Portugal: Tras-os-Montes,Bemposta. AM Bemp	2n = 30	4x	h16		h47		h50, h136,h138–h139, c	h49, h137,h140–h141 c
*B.hybridum*	Bhyb36	Italy: Sardinia. AM Cerdeña	2n = 30	4x		h20				
*B.hybridum*	Bhyb37	Spain: Cazorla, Pto de las Palomas. AM Czpp	2n = 30	4x		h20	h3			
*B.hybridum*	Bhyb38	Turkey: Adiyaman. MT & JV Adi-P1	2n = 30*	4x			h3,h48 c	h20 c		
*B.hybridum*	Bhyb39	Turkey: Balli. MT & JV Bal-P1	2n = 30*	4x			n.a			
*B.hybridum*	Bhyb40	Turkey: HB & JV BdTR6A	2n = 30*	4x		h20		h20	h1,h60 c	h19,h61,h62 c
*B.hybridum*	Bhyb41	Turkey: HB & JV BdTR6B	2n = 30*	4x		h20		h20	h1,h63 c	h64 c
*B.hybridum*	Bhyb42	Israel: Lehavim. AB&SE BRA7	2n = 30*	4x		h20	h14			
*B.hybridum*	Bhyb43	Israel: Ein Fesh’ha. AB&SE BRA9	2n = 30*	4x		h20	h14			
*B.hybridum*	Bhyb44	Israel: Gilbo’a. AB&SE BRA26	2n = 30*	4x		h20	h49			
*B.hybridum*	Bhyb45	Israel: Carmel. AB&SE BRA32	2n = 30*	4x		h20	h13			
*B.hybridum*	Bhyb46	Israel: Ruhama. AB&SE BRA42	2n = 30*	4x		h20			h66 c	h19,h65,h67 c
*B.hybridum*	Bhyb47	Israel: Nahal Bokek. AB&SE BRA57	2n = 30*	4x		h20		h50	h1,h68,h69 c	h19 c
*B.hybridum*	Bhyb48	Israel: Ba’al Hazor. AB&SE BRA117	2n = 30*	4x		h20	h13			
*B.hybridum*	Bhyb49	Israel: Nahal Karkom. AB&SE BRA142	2n = 30*	4x		h20		h20	n.a	n.a
*B.hybridum*	Bhyb50	Israel: Petah Tikwa. AB&SE BRA143	2n = 30*	4x		h20	h3		h1 c	h39,h70,h71,h72 c
*B.hybridum*	Bhyb51	Israel: Dorot. AB&SE BRA144	2n = 30*	4x		h20	h14		h1,h73 c	h39,h74,h75 c
*B.hybridum*	Bhyb52	Israel: Amirim. AB&SE BRA146	2n = 30*	4x		h20	h14			
*B.hybridum*	Bhyb53	Israel: Keshet Cave. AB&SE BRA151	2n = 30*	4x		h20	h14			
*B. hybridum*	Bhyb54	Morocco: El-Ksiba road to Imilchil. RH-JA 176	2n = 30	4x	h5		h1		h12,h13,h14 c	h12,h13 c
*B.hybridum*	Bhyb55	Israel: Tel Fares. AB&SE BRA160	2n = 30*	4x		h20			n.a	n.a
*B.hybridum*	Bhyb56	Israel: Brachyia. AB&SE BRA221	2n = 30*	4x		h20	h51			
*B.hybridum*	Bhyb57	Israel: Ramat Menashe Park. AB&SE BRA245	2n = 30*	4x		h20	h3			
*B.hybridum*	Bhyb58	Israel: Reihan Forest. AB&SE BRA249	2n = 30*	4x		h20	h14			
*B.hybridum*	Bhyb59	Israel: Hadera. AB&SE BRA251	2n = 30*	4x		h27	h3			
*B.hybridum*	Bhyb60	Israel: Golani. AB&SE BRA263	2n = 30*	4x		h20	h52			
*B.hybridum*	Bhyb61	Israel: Sion. AB&SE BRA293	2n = 30*	4x		h20	h13			
*B.hybridum*	Bhyb62	Israel: Tel Aviv Univ, Botanical Garden. AB&SE BRA299	2n = 30*	4x		h20	h3		h76,h77,h78 c	h19 c
*B.hybridum*	Bhyb63	Armenia: Sjunik district; Shvanidzor-Nrnadzor. AC Arm1A	2n = 30	4x		n.a			h1,h80,h82 c	h79,h81 c
*B.hybridum*	Bhyb64	Armenia: Sjunik district; Shvanidzor-Nrnadzor. AC Arm1E	2n = 30	4x		h20	h14			
*B.hybridum*	Bhyb65	Armenia: Sjunik district; Shvanidzor-Nrnadzor. AC Arm1J	2n = 30	4x		h27	h53		h1,h83 c	h39 c
*B.hybridum*	Bhyb66	Armenia: Sjunik district; gorge of Vorotan river. AC Arm2F	2n = 30	4x		h20	h14			
*B.hybridum*	Bhyb67	Armenia: Sjunik district; gorge of Vorotan river. AC Arm2I	2n = 30	4x		h20	h14			
*B.hybridum*	Bhyb68	Turkey: Mugla, Bodrum. RH-JA 4				h20	h14			
*B.hybridum*	Bhyb69	Jordan: Deir ‘Alla, norther Ghor. RH-JA 8	2n = 30	4x		h20	h49		h167,h171, h198c	h18,h84,h85,h86,h87,h88,h89,h90,h91,h150–h166,h168–h170,h172–h197,h199–h200 c
*B.hybridum*	Bhyb70	Jordan: Al-Hemma. RH-JA 12				h20	h13			
*B.hybridum*	Bhyb71	Lebanon: Mohafaza, Casa Jbail, Amshit. RH-JA 17				h20	h14		h92,h93,h94,h96 c	h95 c
*B.hybridum*	Bhyb72	Lebanon: Mohafaza Mt. Lebanon, Ghirfeen. RH-JA 19				h20	h54			
*B.hybridum*	Bhyb73	Lebanon: Mohafaza Batroun Caza, Hannoush. RH-JA 24				h20	h3			
*B.hybridum*	Bhyb74	Lebanon: Mohafaza, Caza, Nahr el Kalb. RH-JA 26	2n = 30	4x		h20	h3			
*B.hybridum*	Bhyb75	Kuwait: Sabyah. RH-JA 56				h20	h3			
*B.hybridum*	Bhyb76	Algeria: Chabet el Akra Gorge, Setif-Bejaia. RH-JA 65				h20				
*B.hybridum*	Bhyb77	Libya: Wadi Cala’a above Ras Hilal. RH-JA 75				h20	h3			
*B.hybridum*	Bhyb78	Morocco: Fes, Ain-Kansera. RH-JA 81				h20	h1			
*B.hybridum*	Bhyb79	Morocco: Taza to Sidi Abdallah, Jbel Tazzeka. RH-JA 85				h20	h13			
*B.hybridum*	Bhyb80	Morocco: Al-Hoceima, Targuist. RH-JA 87	2n = 30	4x		h20	h13		h97 c	h98 c
*B.hybridum*	Bhyb81	Morocco: Xaouen, Bou-Ahmed. Oued Adelmane. RH-JA 88				h20	h55			
*B.hybridum*	Bhyb82	Morocco: Zerhon, Nzaia des beni Ammar. RH-JA 91				h20				
*B.hybridum*	Bhyb83	Morocco: Al-Hoceina, Beni Hassane. RH-JA 94				h20	h13			
*B.hybridum*	Bhyb84	Morocco: Xaouen, Tarsif, Oued-Laou. RH-JA 101				h20	h3		h101 c	h99, h100 c
*B.hybridum*	Bhyb85	Turkmenistan: Kara-Kala. RH-JA 104				h20	h14			
*B.hybridum*	Bhyb86	France: Massif d’ Allauch, Bouches-du Rhône. RH-JA 142	2n = 30	4x		h20			h102 c	h39,h102 c
*B.hybridum*	Bhyb87	Italy: Calabria, Reggio di Calabria. RH-JA 145	2n = 30	4x		h20	h56		h1 c	h103 c
*B.hybridum*	Bhyb88	Greece: Corfu, Cape Sidero. RH-JA 161				h20	h3			
*B.hybridum*	Bhyb89	Morocco: Taza, from Tahala to Tissa. RH-JA 163				h28	h57		h1,h104 c	h19 c
*B.hybridum*	Bhyb90	Morocco: 14 km from Boured. RH-JA 164				h20				
*B.hybridum*	Bhyb91	Morocco: Oujda. RH-JA 170				h20	h13			
*B.hybridum*	Bhyb92	Spain: Mallorca, Pto Pollensa. DL&PC Bdis600*				h20				
*B.hybridum*	Bhyb93	Spain: Mallorca, Pto Pollensa, Formentor. DL&PC Bdis681*	2n = 30*	4x		h20	h1			
*B.hybridum*	Bhyb94	Spain: Mallorca, Pto Pollensa, Mal Pas. DL&PC Bdis701*	2n = 30*	4x		h20	h1			
*B.hybridum*	Bhyb95	Spain: Mallorca, Pto Pollensa, St Vicent. DL&PC Bdis731*	2n = 30*	4x		h20	h1			
*B.hybridum*	Bhyb96	Spain:Mallorca, Pollensa-Soller, Temenia. DL&PC Bdis741*				h20	h1			
*B.hybridum*	Bhyb97	Spain: Mallorca, Escorca, Tossels Cuvert. DL&PC Bdis781*				h20				
*B.hybridum*	Bhyb98	Spain: Mallorca, Escorca, Pico del Tossel. DL&PC Bdis791*				h20	h58			
*B.hybridum*	Bhyb99	Spain: Mallorca, Escorca, Cuvert. DL&PC Bdis811*				h20	h2			
*B.hybridum*	Bhyb100	Spain: Mallorca, Deia. DL&PC: Bdis831*	2n = 30*	4x		h20	h59			
*B.hybridum*	Bhyb101	Spain: Mallorca, Escorca, Sa Calobra. DL&PC Bdis851*	2n = 30*	4x		h20	h60			
*B.hybridum*	Bhyb102	Spain: Mallorca, Paguera, Cala Fornell. DL&PC Bdis871*	2n = 30*	4x		h20	h1			
*B.hybridum*	Bhyb103	Spain: Mallorca, Valldemosa, Sa Marina. DL&PC Bdis931*				h20	h61			
*B.hybridum*	Bhyb104	Spain: Menorca, Foruell, Torre Foruell. DL&PC Bdis1001*				h20	h3			
*B.hybridum*	Bhyb105	Spain: Menorca, Ciutadella, Port. DL&PC Bdis1011*	2n = 30*	4x		h20		h62		
*B.hybridum*	Bhyb106	Spain: Mallorca, Llucmajor, Cap Blanc. DL&PC Bdis1031*				h20	h2			
*B.hybridum*	Bhyb107	Spain: Mallorca, Les Salines. DL&PC Bdis1051*				h20	h63			
*B.hybridum*	Bhyb108	Spain: Mallorca, Santanyi, Parc Natural. DL&PC Bdis1071*				h20	h64			
*B.hybridum*	Bhyb109	Spain: Mallorca, Llucmajor, Algaida. DL&PC Bdis1101*				h20	h65			
*B.hybridum*	Bhyb110	Spain: Mallorca, Capdepeva, Mesquida. DL&PC Bdis1131*	2n = 30*	4x		h20	h1			
*B.hybridum*	Bhyb111	Spain: Mallorca, Pollensa, P. Margalida. DL&PC Bdis1191*	2n = 30*	4x		n.a	h1			

Asterisks indicate chromosome counts obtained in this study using DAPI-staining methods. Other chromosome numbers and/or ploidy levels were retrieved from the literature or have been provided by the germplasm collectors and curators (see abbreviations below). For each sample, the sequences have been classified into the corresponding *trn*LF, ITS and GI haplotypes (see Table S2). Bdis and Bsta indicate *B. distachyon*-type and *B. stacei*-type sequences, respectively. c: cloned sequences; n.a: not amplified. Abbreviations of germplasm and sample collectors and curators, germplasm banks and herbaria: AB & SE: Adina Breiman and Smadar Ezrati; ABR: Aberystwyth (UK); AC: Ana Caicedo; AM: Antonio Manzaneda; DL & PC: Diana López-Alvarez & Pilar Catalán; EB: Elena Benavente; CS: Consuelo Soler; Hikmet Budak: HB; INIA-CRF: Instituto Nacional de Investigaciones Agrarias - Centro de Recursos Fitogenéticos (Spain); INRA: Institut National de la Recherche Agronomique (France)**;** JV: John Vogel; MA: Real Jardin Botanico de Madrid herbarium; Metin Tuna: MT; MRR: Mohammad Reza Rahiminejad; PC & LM: Pilar Catalan and Luis Mur; RH-JA: (Univ.) Reading Herbarium - Joel Allainguillaume; SH: Samuel Hazen; ST: Salvador Talavera; USDA: United States Department of Agriculture. Literature references for chromosome counts and ploidy levels: Jenkins et al. (2003), Filiz et al. (2009); Catalán et al. (2012), Giraldo et al. (2012); Hammami et al. (2011), Manzaneda et al. (2011); Mur et al. (2011); Vogel et al. (2009).

**Table 2:** Sequence variation and discrimination power of the three studied loci for the DNA barcoding of the *Brachypodium distachyon* complex taxa (*B. distachyon*, *B. stacei*, *B. hybridum*).

	*trn*LF		ITS		GI
Number of species	3		3		3
Number of sequences (including clones)	204		197 (281)		56 (342)
Number of aligned nucleotide sites	782		612		665
% amplification sucess	98		91.5		98
% sequencing success	92.9		97.5		98
% species sucessfully identified (*Bdistachyon*/*Bstacei*/*Bhybridum*)	100/100/96.3		100/100/75		100/100/100
% Variable nucleotide sites	4.7		15.3		39.5
% Diagnostic nucleotide sites	3.2		7.3		11.3
Overall mean intraspecific distance (diploid species) (min-max)	0.011 (0–0.028)		0.029 (0–0.075)		0.022 (0–0.042)
*B. distachyon* mean intraspecific distance (min-max)	0.005 (0–0.013)		0.005 (0–0.010)		0.004 (0.002–0.006)
*B. stacei* mean intraspecific distance (min-max)	0.001 (0–0.003)		0.005 (0–0.017)		0.003 (0–0.006)
*B. hybridum* (*B. distachyon*-like) mean intraspecific distance (min-max)	0 (0–0)		0.010 (0–0.043)		0.008 (0–0.046)
*B. hybridum* (*B. stacei*-like) mean intraspecific distance (min-max)	0.003 (0–0.005)		0.013(0.002–0.032)		0.011 (0–0.046)
Mean interspecific distance between *B.distachyon* - *B. stacei* (sd)	0.024 (0.005)		0.055 (0.009)		0.038 (0.007)
Mean interspecific distance between *B.distachyon* - *B. hybridum* (*B.distachyon*-like) (sd)	0.009 (0.003)		0.008 (0.001)		0.006(0.001)
Mean interspecific distance between *B. stacei* - *B. hybridum* (*B.stacei*-like) (sd)	0.002(0.001)	0.009 (0.002)		0.007 (0.001)	
Mean interspecific distance between *B. hybridum* (*B.distachyon*-like) - *B. hybridum* (*B.stacei*-like) (sd)	0.021(0.005)		0.054 (0.009)		0.040 (0.007)

*B. distachyon*- and *B. stacei*-like refer to the *B. hybridum* sequences inherited, respectively, from one or the other parent. sd, standard deviation.

**Figure 2:**
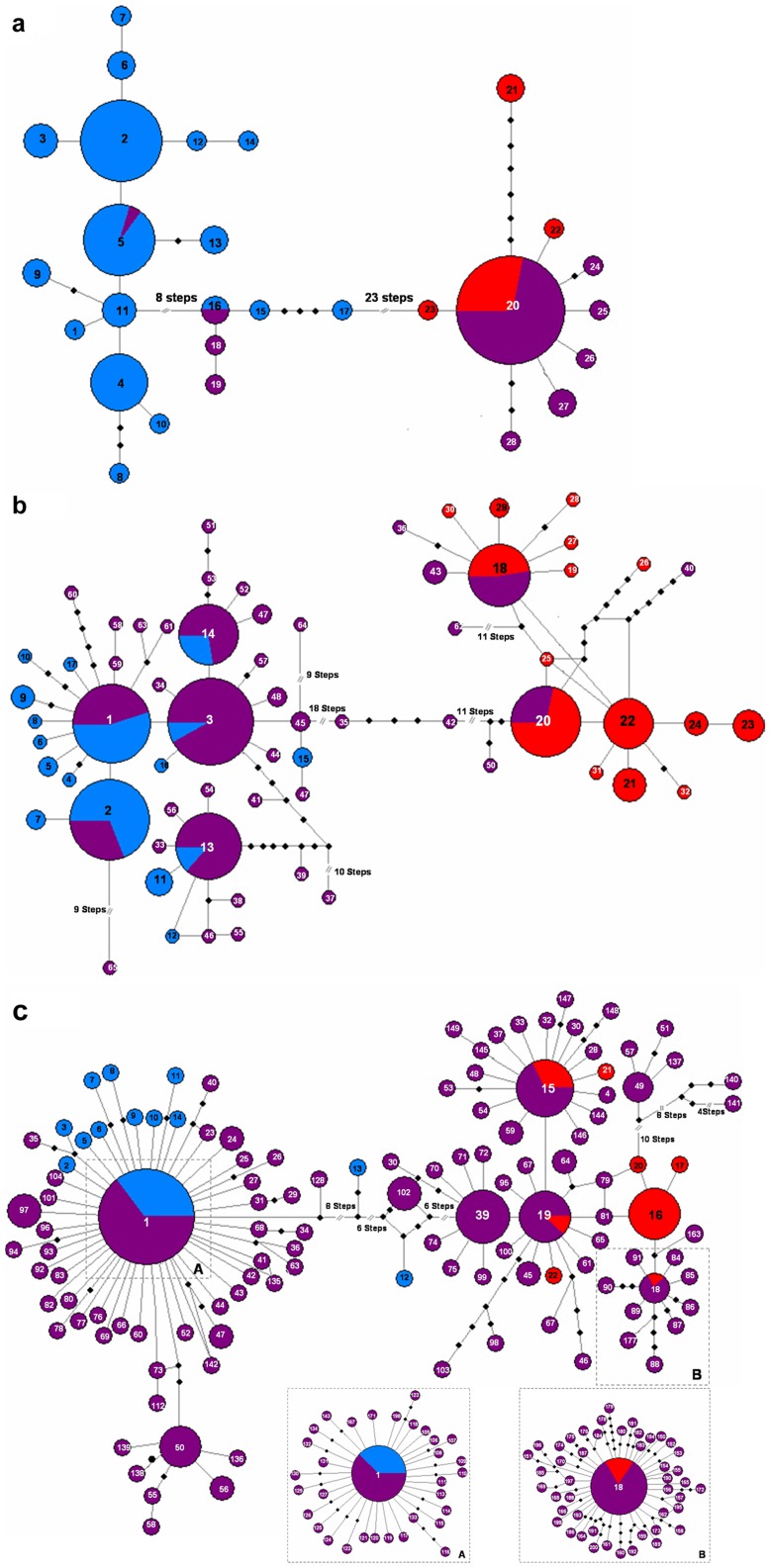
Haplotype networks of the *Brachypodium distachyon* s. l. taxa (*B. distachyon* (blue), *B. stacei* (red) and *B. hybridum* (purple) constructed from DNA sequences of each of the three studied barcoding loci using statistical parsimony methods. a) *trn*LF network; b) ITS network; c) GI network (boxes A and B show additional *B. distachyon*-type and *B. stacei*-type haplotypes, respectively). Each haplotype is represented by a circle with size proportional to the number of sequences that share the haplotype. Haplotype numbers correspond to those indicated in Tables 1 and S2. Dots indicate missing haplotypes.

**Figure 3:**
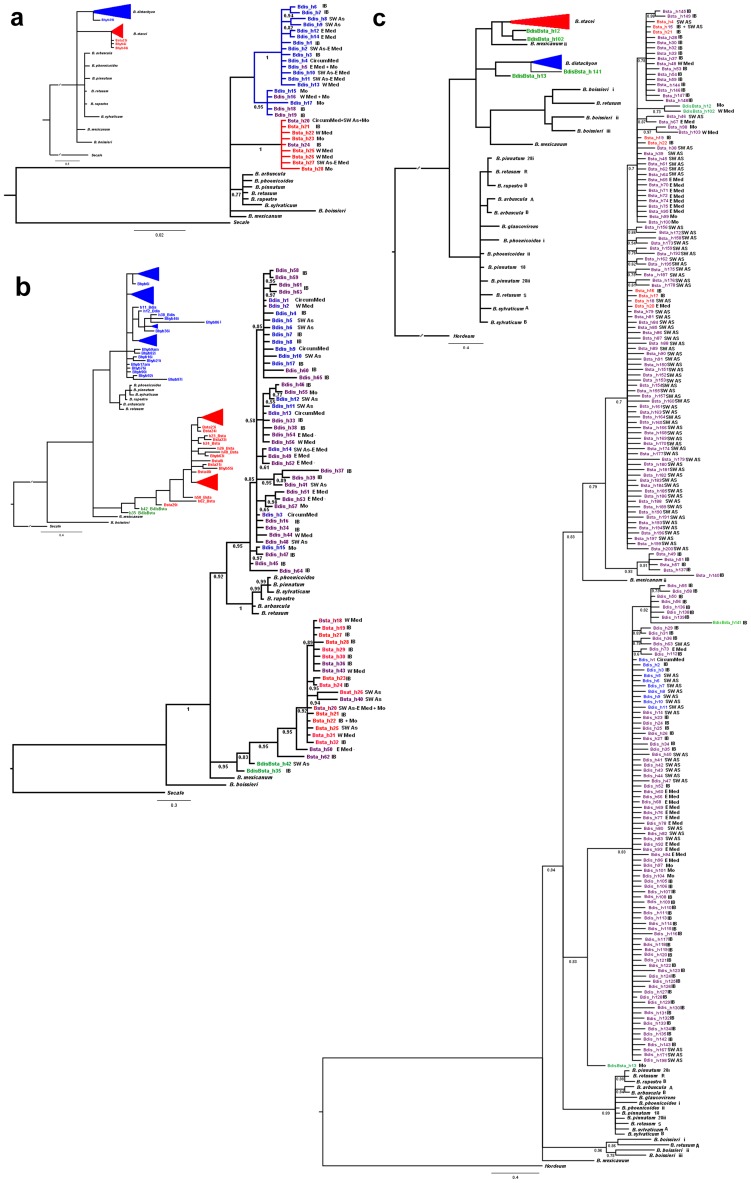
Bayesian halfcompat consensus trees of the *Brachypodium distachyon* s. l. taxa (*B. distachyon* (blue), *B. stacei* (red) and *B. hybridum* (purple) based on analysis of DNA sequences. a) *trn*LF tree; b) ITS tree; c) GI tree. *B. distachyon*-type and *B. stacei*-type clades are shown as blue and red triangles, respectively, in the small subfigures; potential recombinant parental sequences of *B. hybridum* (BdisBsta, see Table S2) are indicated in green. ‘i’ and ‘am’ indicate, respectively, incomplete and ambiguous sequences. Numbers below branches correspond to posterior probability support (PPS) values above 0.5. Geographical distributions of sequenced samples are indicated in the large subfigures (CircumMed - circumMediterranean; E Med - eastern Mediterranean; IB - Iberian Peninsula; Mo - Morocco; SW As - southwestern Asia; W Med - western Mediterranean).

The aligned *trn*LF region of *B. distachyon* – *B. stacei* – *B. hybridum* sequences consisted of 782 nucleotide positions of which 38 (4.9%) were variable and 25 (3.2%) were potentially informative (Tables 2, S2, S3). In total, 28 *trn*LF haplotypes were found (Tables 1, S2); these were classified as *B. distachyon*-type (h1 - h19) and *B. stacei*-type (h20 - h28) haplotypes. The *B. distachyon-*type and *B. stacei*-type clusters of haplotypes were monophyletic with respect to one another (Figs. 2, 3). Most of the *B. hybridum trn*LF sequences were shared with or derived from *B. stacei*-type sequences (n = 102; 96.2%) and only a few of them came or were derived from *B. distachyon*-type ones (n = 4; 3.8%) (Table 1). The most common haplotype overall (h20) was shared by most of the *B. stacei* and *B. hybridum* sequences, whereas the *B. distachyon* sequences were partitioned into three main haplotypes (h2, h5, h4) and several minor ones (Table S2).

The aligned ITS region had a length of 612 nucleotide positions of which 105 (17.2%) were variable and 43 (7.0%) were potentially informative (Tables 2, S3). The complete ITS data matrix of unambiguous direct or cloned sequences distinguished 65 ITS haplotypes (Tables 1, S2). The *B. distachyon*-type haplotypes (n = 43, 66.2%) outnumbered the *B. stacei*-type (n = 19, 29.2%) ones. There were 5 (h1-h3, h13-h14) and 3 (h18, h20, h22) main groups of haplotypes in each respective class, in terms of frequency among the total sample set; the remaining haplotypes mostly corresponded to single-individual or single-clone haplotypes. The *B. distachyon-*type and *B. stacei*-type clusters of haplotypes were monophyletic with respect to one another (Figs. 2, 3). Though most of the *B. distachyon* and *B. stacei* clones sequenced were identical within individuals, some gave different haplotypes (e. g. Bdis8, Bdis36, Bsta1, Bsta5, Bsta7, Bsta24; Tables 1, S2). Most of the *B. hybridum* ITS sequences were similar to the *B. distachyon*-like parental ones (n = 78; 83%); however few of them were similar to the *B. stacei*-like ones (n = 6; 6.4%), and several of them still showed similarities to both parental copies (n = 10; 10.6%) (Tables 1, S2). A very low percentage of the co-inherited ITS sequences showed evidence of inter-parental sequence recombination in *B. hybridum* (4.6%).

The aligned GI region consisted of 665 nucleotide positions of which 146 (21.9%) were variable and 45 (6.8%) were potentially informative (Tables 2, S3). The GI sequences were more variable than those of either *trnLF* or ITS, grouping into 200 haplotypes of which approximately the same number were of *B. distachyon* (n = 90, 45%) and *B. stacei*-type (n = 106, 53%) (Tables 1, S2). These two groups were monophyletic with respect to one another (Figs. 2,3). The few cloned *B. distachyon* and *B. stacei* individuals showed GI haplotypes belonging to their respective groups but with slightly different allelic variants in most cases. These minor variants could represent genuine mutations but could be also a consequence of Taq polymerase errors (Harriet Hunt, pers. comm.). Four haplotypes (h12, h13, h102, h141) showed evidence of inter-parental recombination in *B. hybridum* (n = 4, 2%) (Tables 1, S2). Most (n = 29, 96.7%) of the studied *B. hybridum* individuals showed two types of GI sequence, one type of which was inherited from each of the two parental species (Table 2); however the number of clones inherited from one or the other parent was dissimilar in some cases and, in only one instance, all of them were from a single parent (n = 1, 3.3% ) (Tables 2, S2).

K2P pairwise substitution rates, the recommended standard distance model in barcoding studies [32], [33], showed high interspecific sequence divergence values and low intraspecific values between and among the diploids *B. distachyon* and *B. stacei* for the three analysed data sets (Table 2). Both the mean intra- and interspecific divergence values were higher for the more variable nuclear ITS (0.029 (2.9%) and 0.055 (5.5%) respectively) and GI loci (0.022 (2.2%) and 0.038 (3.8%) respectively) than for the more conserved plastid *trn*LF locus (0.011 (1.1%) and 0.024 (2.4%) respectively). Moreover, the percentage of correctly identified specimens of a given species was in all cases above the 50% cut-off threshold suggested as a baseline to discriminate among species [21] (*trn*LF: 100/100%; ITS: 100/100%; GI: 100/100%; for *B. distachyon* and *B. stacei*, respectively). This supported the existence of a typical barcode gap for *B. distachyon* and *B. stacei* in all the three loci. Regarding *B. hybridum*, the K2P “intraspecific” and “interspecific” divergence rate calculations, conducted separately with respect to their two parental-donor sequences, showed sequence divergence values similar to those found in *B. distachyon* and *B. stacei* for the three loci (Table 2). The differences between the intra-parental and inter-parental (*B. distachyon*-like vs *B. stacei*-like) mean values were equivalent to those found between and within the sequences of the two diploids, and the barcoding gaps were also present in all three loci (Table 2). The percentage of individuals known from cytogenetic data to be *B. hybridum*, which showed the expected *B. hybridum* signature in the sequence data, was >50% with the use of either the combined *trn*LF+ITS core (90%) or the GI (96.7%) sequences (Tables 1, S2). We could therefore equate these values to the respective percentages of correct identification obtained from one and the other data set.

Haplotype networks constructed for each of the separate data sets using statistical parsimony methods (Fig. 2) showed a clear-cut separation between the *B. distachyon*-type and *B. stacei*-type classes of sequences in all cases. The plastid *trn*LF network required a connection of 23 steps between the two main haplogroups (Fig. 2a). The commonest *B. stacei*-type, h20, included *B. stacei* and *B. hybridum* individuals spread across all the SW Asian-Mediterranean and Macaronesian region (and also the respective type specimens of *B. stacei* (Spain: Formentera; ABR114) and *B. hybridum* (Portugal: Lisbon; ABR113). Its satellite haplotypes (h22–h28) corresponded to *B. stacei* and *B. hybridum* individuals from distinct western and eastern Mediterranean localities; the most isolated, h21 (6 step connection) was shared by individuals from Eastern Spain and the Balearic Islands (Tables 1, S1; Fig. 2a). The *B. distachyon*-type network was more diverse, with haplotypes separated by several steps and containing almost exclusively *B. distachyon* individuals (Fig. 2a). The core-group was formed by three main haplotypes, the interconnected h2, h5 and h4, which were found in individuals from disparate Mediterranean localities, plus the *B. distachyon* type (Iraq, Bd21; h2).

The nuclear ITS network was more complex than the *trn*LF one; however, it also distinguished two highly divergent *B. distachyon*- and *B. stacei*-type clusters that were separated by 33 steps (Fig. 2b). These clusters were linked by two intermediate haplotypes (h35, h42) from *B. hybridum* individuals from both sides of the Mediterranean that likely corresponded to inter-parental recombinant sequences (Table S2). The *B. stacei* cluster showed three main haplotypes interconnected by single steps (Fig. 2b). One haplotype (h19) comprised *B. stacei* and *B. hybridum* individuals from across the Mediterranean region and the Canary Islands, including most of the clones of the *B. stacei* type specimen (ABR114). The other two main haplotypes mostly comprised eastern Mediterranean (h20) or exclusively western Mediterranean (h22) samples (Tables 1, S2). Among the satellite haplotypes of the latter group, close phylogeographic connections were also detected between E Spain and the Balearic Islands (h23 and h24). The group showed a pattern of few unresolved loops, likely caused by intraindividual or by intraspecific *B. stacei*-type sequence recombinations. The more diverse *B. distachyon* cluster contained five main haplotypes, four of them interconnected by single mutations (h1, h2, h3, h14) and a fifth one (h13) nested within a derived 14-step subcluster (Fig. 2b). Haplotypes h1, h3, and h13 included *B. distachyon* and *B. hybridum* individuals from across the Mediterranean region and the first also included the *B. distachyon* type specimen (Bd21). However, h2 and h14 were more structured geographically, containing only Iberian-Balearic or mostly SW Asian-E Mediterranean individuals, respectively. The *B. hybridum* type specimen (ABR113) sequences were divided among two haplotypes (h38, h29). The *B. distachyon* cluster also showed one loop within the h13 subcluster, though the remaining satellite haplotypes were connected linearly, with different numbers of stepwise mutations (Fig. 2b).

The level of diversity and complexity was higher in the GI network (Fig. 2c); nonetheless it also showed a clear-cut split between the *B. distachyon-* and *B. stacei*-type clusters that required a connection of 30 steps. Two kinds of potential interspecific recombinant haplotypes, closer to either the *B. distachyon* (h13) or the *B. stacei* (h12, h102) clusters, were observed between them (see also Fig. 3c). Within the *B. distachyon* cluster, the commonest haplotype, h1, included *B. distachyon* and *B. hybridum* individuals from across the Mediterranean region (including the *B. distachyon* (Bd21) and *B. hybridum* (ABR113; *B. distachyon*-like copy) type specimens). Most of the h1 satellite haplotypes differed by one or two stepwise mutations; however, a more distantly related subclade was also present, formed exclusively of Iberian haplotypes (h50, h55-h56, h58, h136, h138, h139). Four unresolved loops involving *B. hybridum* haplotypes indicated the likely occurrence of intraspecific *B. distachyon*-like sequence recombinations in the hybrids. The *B. stacei* cluster comprised four main haplotypes. Two of them, h15 (including the *B. stacei* (ABR114) and the *B. hybridum* (ABR113; *B. stacei*-like copy) type specimens) and h19, included individuals of both species from the whole Mediterranean region. A third one, h39, comprised only *B. hybridum* individuals mostly from the eastern Mediterranean region. In contrast, the more derived h16 comprised only *B. stacei* individuals from the Iberian Peninsula. An isolated subcluster (separated by 10 steps) was formed by six haplotypes (h49, h51, h57, h137, h140, h141) from southern Spain. The *B. stacei* group showed a more intricate pattern of loops and divergences among the haplotypes than that of the *B. distachyon* group (Fig. 2B), likely reflecting a more complex evolutionary history.

The NJ trees based on K2P distances (Fig. S1) reflected the above findings and their topologies were highly congruent with the Bayesian halfcompat consensus trees shown here. In the *trn*LF tree (Fig. 3a) the *B. distachyon* and *B. stacei* sequences fell into two separate fully supported clades (1.00 posterior probability support); these clades collapsed into a polytomy with the core-perennial clade, *B. boissieri* and *B. mexicanum*. The nine haplotypes of the *B. stacei* clade were unresolved; however, the 19 haplotypes of the *B. distachyon* clade split into two strongly supported clades. One of them included the 5 divergent haplotypes of intermediate placement in the haplotype network (Fig. 2a), which are mostly distributed in the western Mediterranean region, and the other included the majority of the remaining haplotypes (Fig. 3a). Within this second group, some resolution was obtained for three separate Iberian (0.94), Turkish (0.82) and Middle East (0.98) subclades. The ITS tree depicted a strong divergence of the highly supported *B. stacei* (0.95) and *B. distachyon* (0.85) clades (Fig. 3b); *B. stacei* was unresolved in a sub-basal position with *B. mexicanum*, whereas *B. distachyon* was resolved as sister to the core perennials clade (0.92). The internal resolution of both clades was poor; however, two separate eastern Spain/Balearic Islands (0.95) and Iranian (0.94) subclades and two Balearic Islands (0.97, 0.95) subclades were recovered within, respectively, the *B. stacei* and *B. distachyon* clades. The GI tree also supported the divergent history of the *B. distachyon* (0.99) and *B. stacei* (0.99) lineages (Fig. 3c). *B. stacei* was sister to *B. mexicanum* p. p. (0.99), whereas *B. distachyon* was unresolved with respect to the weakly supported *B. boissieri-B. retusum*/*B. mexicanum* p.p. clade. The resolution within the *B. distachyon* clade was low except for a well supported (0.99) Iberian subclade that corresponded to the isolated subcluster of southern Spain *B. hybridum* (*B. distachyon*-like) haplotypes (h50, h55, h56, h58) detected in the network (Fig. 2c). Similarly, the *B. stacei* clade split into two well supported subclades, one of which also corresponded to a subcluster of highly isolated southern Spain *B. hybridum* (*B. stacei-* like) haplotypes (h49, h51, h57) recovered in the network (Fig. 2c; Tables 2, S1).

## Discussion

### DNA Barcodes for *B. distachyon*, *B. stacei* and *B. hybridum*

Under the premise that a successful barcode locus should enable the recovery of monophyletic clusters corresponding to individual species [34], we found that any one of the three assayed loci (*trn*LF, ITS, GI) could unambiguously differentiate the two monophyletic diploid species from direct sequencing of PCR amplicons. However the identity of the allotetraploid requires combined analysis of direct *trn*LF and direct or cloned ITS sequences or through analysis of cloned GI sequences.

Our results demonstrate that the widely employed barcoding regions *trn*LF and ITS [20], [23] clearly discriminate between *B. distachyon* and *B. stacei*. Both regions showed: i) high inter- vs intraspecific distance divergences, ii) significant barcoding gaps (Table 2), iii) extremely distant monophyletic clusters in the parsimony networks (Figs. 2a, b); and iv) highly supported divergent monophyletic clades in both the NJ (Results not shown) and the Bayesian trees (Figs. 3a, b). They also comply with the requirements of feasibility and rapid and easy production of the sequences to be considered optimal barcoding molecules [20]. However, the allopolyploid nature of *B. hybridum*, together with its estimated recent origin (c. 1 Ma; [11]), prevents their direct use as single standard barcodes for this taxon and its two parental taxa. Our study has shown that the maternally-inherited *B. hybridum trn*LF haplotype sequences could have been acquired from either of the two parents (Table 1; Figs. 2a, 3a) and that the biparentally-inherited *B. hybridum* ITS copies (*B. distachyon*-like and *B. stacei*-like) could either have remained intact in the hybrid genome or could have converged into one or the other parental copy (Table 1, Figs. 2b, 3b). This creates the possibility of misleading results if the *B. hybridum trn*LF and ITS sequences had been respectively inherited from and (co-inherited but) converted into the same progenitor sequences, causing confusion between the parent and the allotetraploid taxa (e. g. Bhyb26, Bhyb30and Bhyb35 with *B. distachyon*, and Bhyb28, Bhyb40, Bhyb41, Bhyb 47, Bhyb49 and Bhyb105 with *B. stacei*; Table 1). Cloning of the ITS sequences can help to solve the uncertainty if both parental copies are detected, as demonstrated in several studied cases (e. g. Bhyb9, Bhyb10, Bhyb14, Bhyb15, Bhyb18, Bhyb19, Bhyb22, Bhyb23 and Bhyb38; Table 1). The use of the combined *trn*LF+ITS barcode shows high percentages of successful species discrimination among the species in the reticulate triangle using either direct *trn*LF and ITS sequences (93.3%) or direct *trn*LF and cloned ITS sequences (94% (Tables 1, S2). The barcoding would remain untractable, however, if the concerted-evolution mechanism that operates in the multicopy nuclear ribosomal genes [26], [35] had converted all the co-inherited copies into the same parental copy.

Because of the drawbacks posed by the use of these classical barcodes, we searched for an alternative nuclear locus that could unambiguously differentiate the three species. This could only be a single-copy nuclear gene that retained both parental copies in the allotetraploid without undergoing convergent evolution towards one of them. Among the several COS proposed as appropriate candidates to differentiate closely related plant species [28], [29], [30] and to discriminate among *Brachypodium* taxa [11], [17] we selected a 665 bp fragment of the *GIGANTEA* gene, one of the key regulators of flowering promotion and phase transition [36]. This GI region has proved to be a strong candidate barcode for the *B. distachyon* s. l. taxa based on: i) its easy amplification, cloning and sequencing; ii) its single-copy orthologous nature: iii) the accumulation of discriminating mutations between the *B. distachyon* and *B. stacei* sequences (3.8% of mean inter- vs. intraspecific distance divergence and a significant barcode gap, Table 2); iv) the common presence of the two different co-inherited parental *B. distachyon*-like and *B. stacei*-like GI sequences in *B. hybridum* (Table 1); and v) rarely, the presence of inter-parental recombinant sequences that could be easily detected (Table1). The genetic differences were reflected in the GI parsimony network (Fig. 2c) and in the NJ (Results not shown) and Bayesian GI (Fig. 3c) topologies that recovered, respectively, distant clusters and well supported divergent monophyletic clades for *B. distachyon* and *B. stacei*, each of them including their respective derived *B. hybridum* copies. Although 5 cloned sequences were sufficient to detect both parental copies in most of the studied *B. hybridum* samples, a few difficult samples required the screening of up to 10–16 clones (e. g. Bhyb13, Bhyb34, Bhyb35 Bhyb50) or even a larger number, like in the case of the Bhyb69 sample (58 clones), to pick up variation from both parental species. Nonetheless, one sample (Bhyb2) showed only one parental copy after a relatively intensive clonal screening (49 clones; Tables 1, S2). This implies that a larger number of GI clones should be sequenced in order to detect co-inherited copies from both parents, providing that they are still maintained in the hybrid genome.

All the above evidence supports the choice of the GI locus as an alternative or as an additional suitable barcode for discriminating among the triangle species of the *B. distachyon* s. l. complex. This demands the use of cloning procedures but reduces the number of surveyed loci to just one. Moreover, the percentage of successful species discrimination increases to 98.2% (Tables 1, S2), which is above than that of the combined *trn*LF+ITS barcode. It further complements alternative cytogenetic identifications based on genome size or chromosome counting. The choice of the best method in a given situation would depend on considerations of facilities and costs, the acceptable error rate, and a priori information on the levels of polyploids in the sample. Very likely, other single-copy genes, such as those analysed within *Brachypodium* that also showed both co-inherited parental copies in the derived hybrid (e. g. CAL, DGAT, SST3; [11], [17]), could also serve as barcodes for this group of taxa. Single-copy nuclear genes are not ideal universal barcodes for plants as their priming sites cannot be easily transferred to non-related groups (e. g. [37]). The GI locus has been successfully amplified and sequenced in different representatives of Pooideae (López-Álvarez & Catalán, unpublished data) and could probably be extended to all the grass family. We propose the use of single-copy genes as a suitable barcoding alternative to circumvent the problem posed by the existence of recently evolved hybrids and polyploids within specific plant groups. In the future, the use of Next Generation Sequencing (NGS) data (e. g. [38], [39], [40]), may facilitate the barcoding of problematic plant groups which contain recently evolved hybrids and polyploids. Although the availability of NGS data is still limited both taxonomically and among laboratories, its use for this purpose is rapidly increasing. In the mean time, the use of single-copy genes is the most practicable current solution for barcoding such plant groups.

### Utility of the Proposed Barcoding Method

The new DNA barcoding method proposed here has direct applications to many on-going studies of the model plant *B. distachyon* and its close allies [2], [4]. It has great relevance to the selection of wild germplasm for genomic (http://brachypodium.pw.usda.gov) and plant breeding programs, and for ecological and evolutionary studies of wild populations [6], [11]. For this, the correct identification of the three species is crucial but still troublesome due to uncertainty in identifications based on highly variable morphological traits and on ambiguous genome sizes, which show overlapping sizes for *B. distachyon* and *B. stacei* [11], [41]. Our study has revealed several misidentifications of *B. distachyon* and its close relatives *B. stacei* and *B. hybridum* in germplasm collections (e. g. USDA, ABR) and inbred lines (cf. [7], [8], [11], [42]; e. g., Bsta9, Bsta42, Bsta43, Bhyb9, Bhyb10, Bhyb19, Bhyb20, Bhyb21, Bhyb38, Bhyb39, see Table 1) that likely resulted from incorrect orcein-staining chromosome counts or misleading genome size measurements. Alternatively, the misidentifications could also result from the mixed sampling of individuals or seeds of different species from admixed populations. This problem has been manifested in the failure of ‘intraspecific’ *B. distachyon* crossing programs, which were in fact interspecific (Magda Opanowicz and John Doonan, pers. comm.) and in unexpected results from cell wall analyses of putative *B. distachyon* lines, which corresponded to *B. stacei* or *B. hybridum* lines (Richard Sibout, pers. comm.). Our barcoding method overcomes these problems, providing an efficient and automatable method to discriminate among the three species.

The validity of our proposed barcoding method depends on the large genetic divergences detected between the diploid *B. distachyon* and *B. stacei* genomes for the three analysed loci (Tables 1, 2). The high number of synapomorphic mutations separating them (23, 33, and 30, respectively, for the *trn*LF, ITS and GI loci; Fig. 2), facilitates the immediate classification of the genomes, even from incomplete sequences (Figs. 3a, b, c). Furthermore, the three loci provide informative indels that differentiated *B. distachyon* and *B. stacei*, like the two 6-bp gaps in the *trn*LF locus, the two 3- and 4-nts gaps in the ITS locus, and the one 1-nt gap in the GI locus (see Table S3). Within the ITS region, the ITS2 spacer covers the two diagnostic indels and more than half of the synapomorphic markers detected within the locus (24 out of 43; Table S3), supporting the proposal that the ITS2 subregion could be used alone (Hollingstworth 2011) to barcode case study species. The correct identification of *B. hybridum* would always require, however, the combined use of, at least, the *trn*LF+ITS barcoding sequences. Our data indicate that direct PCR sequences from the two genes could discriminate *B. hybridum* from its two parental species in a high percentage of the cases (88.75%; Table 1). This value increases to 90.0% when the ITS products are cloned. However, as the method might not permit full resolution, due to the potential inheritance of the same parental plastid *trn*LF and converted nuclear ITS sequences in the hybrid (cf. [25]), the single-copy GI locus was selected as an alternative barcode for the species in the triangle. The random screening of 5 individual GI clones gave a relatively high resolution (80%) that became higher (96.7%) when up to 10–16 (and exceptionally more, e. g. 58) clones were sequenced within our surveyed samples (Table 1).

Recently, Giraldo et al. (2012) [41] proposed a new molecular method to differentiate the three taxa based on the different allelic SSR profiles of *B. distachyon* and *B. stacei* at four nuclear microsatellite loci and their additive patterns in *B. hybridum*. This represents an important step forward for rapid molecular identification of the species, similar to the molecular marker-based barcoding methods proposed for taxonomically complex and highly reticulate plant (e. g. [43]) and animal (e. g. [44]) groups. However, these methods could be less stable and prone to substantial changes than the sequence-based ones as the SSR allelic variation of the barcoded species might be greater than their DNA sequences (and consequently overlap) when a wider range of samples is used [45]. The discriminating SSR markers proposed by Giraldo et al. (2012) [41] were tested across a wide representation of Spanish samples and in the type specimens of the three taxa, but they were not studied in samples from other Mediterranean regions. Thus, our barcoding approach and that of Giraldo et al. (2012) [41] could be used in a complementary way (e. g. [44]) for rapid and accurate molecular identification of the ‘Brachy-complex’ taxa [2], allowing for confident identification even when unusual allelic variation renders one or other method unreliable.

### Genetics and Geographical Distributions of the Three Species and the Polyphyletic Origin of *B. hybridum*

Our current barcoding survey of *B. distachyon*, *B. stacei* and *B. hybridum* samples has encompassed the whole Mediterranean region, the native distribution area of the three species [11]. One of the main findings of the study is the detection of *B. stacei* populations in both the western and eastern Mediterranean regions (Table 1; Fig. 1). This rare species was until recently only known from the type locality (Spain: Balearic Islands: Formentera) [11]. However, other recent studies have indicated its presence in other localities of SE Spain [7], [41] and in the Canary Islands [41]. Our analyses have confirmed most of these findings and have also revealed its presence in other western Mediterranean localities (Mallorca (Balearic Islands), S Spain, NW Morocco; Table 1, Fig. 1) where it was mislabelled as *B. distachyon* in the herbaria vouchers. Most notably, we have revealed the presence of *B. stacei* in the SW Asian-Middle East region (Iran, Israel, Lebanon, Palestine; Table 1), from which it was unknown and also misclassified as *B. distachyon*. Knowledge of this broader native geographical distribution area of *B. stacei* will be highly valuable for the selection of new ecotypes and local lines that could be used in the generation of F2 progenies to help the assembly of the newly sequenced *B. stacei* ABR114 genome (http://brachypodium.pw.usda.gov; John Vogel, pers. comm.). Our study has also contributed to understanding the native distribution areas of the more widely distributed species *B. distachyon* and *B. hybridum* (Table 1; Fig. 1). Both taxa are widespread in the Mediterranean region and largely overlap [2], [8], [11]. The new barcoding data confirm their presence on both sides of the Mediterranean basin, from which regions most the germplasm lines have been generated [2], [8], [41], and also report their presence in the central Mediterranean area (Table 1). This would be also a valuable source of information for the selection of new *B. hybridum* ecotypes and lines for the production of F2 progenies that would complement the assembly of the newly sequenced *B. hybridum* ABR113 genome (http://brachypodium.pw.usda.gov; John Vogel, pers. comm.), and those of *B. distachyon* that could be added to the resequencing project of the model plant.

Despite their abundant distributions in the Mediterranean, the intraspecific genetic diversities of the parental *B. distachyon* (0.5% *trn*LF and ITS; 0.4%GI) and *B. stacei* (0.1% *trn*LF; 0.5% ITS; 0.3% GI) sequences were low (Table 2). This was manifested in the sharing of their respective most common *trn*LF, ITS and GI haplotypes by individuals from populations located far apart in the circumMediterranean region (Tables 1, S2; Fig. 2). In contrast, individuals from geographically close populations, or even intraindividual clones, showed different haplotypes. Our results agree with those of Vogel and co-workers [8] and Mur and co-workers [2], based on SSR markers, which found close genetic connections between geographically distant *B. distachyon* populations in Turkey and between Spain and Turkey, respectively. Selfing species are expected to show low within-population and high among-population genetic diversities [46]. However, the autogamous *B. distachyon* and *B. stacei* samples show low overall geographical structuring of genetic diversity. This might be a consequence of the long distance dispersal of their seeds (cf. [8]) and the high capability of these annuals to adapt to different environmental conditions (cf. [6]). The genetic diversity of the less abundant *B. stacei* could be lower than that of the more widespread *B. distachyon*, as deduced from the proportionally fewer *trn*LF and ITS haplotypes detected in the former (Table 1). Both taxa show, however, some traces of geographic isolation between the western and eastern Mediterranean regions, evidenced by the detection of regional haplotypic clades (e. g. B. *distachyon*: western Mediterranean, Iberian, Turkish and Middle East subclades (*trn*LF, Fig. 3a); *B. stacei*: E Iberian-Balearic and Turkish subclades (ITS, Fig. 3b). The phylogeographic study of these populations is currently in progress (López-Álvarez and coauthors, unpublished results).

Another striking finding of our study is the demonstration of the existence of different directional crosses that likely gave rise to the new allotetraploid species (Tables 1, S2; Figs. 3a, b, c). In the more restricted study of Catalán and co-workers [11], all the surveyed *B. hybridum* individuals showed the inheritance of a *B. stacei*-like plastid genome, resulting from a cross with maternal *B. stacei* and paternal *B. distachyon* parents. However, our survey with larger sample sizes shows that, although the above seems to be most common cross direction, in a few cases the *B. hybridum* individuals are derived from a cross between maternal *B. distachyon* and paternal *B. stacei* parents (Table 2; Fig. 3a). The fact that *B. hybridum* plants derived from the alternate-direction crosses occurred in different Mediterranean localities (Table 1; Fig. 1) supports the multiple and polytopic origins of the allotetraploid *B. hybridum*. A closer inspection of the more variable ITS and GI networks and phylogenetic trees also reveals distinct relationships of the *B. hybridum* sequences to different parental haplotypic groups (Table 1; Figs. 2, 3) corroborating the polyphyletic origin of the *B. hybridum* samples. Complementary or unique parental haplotypic clusters have been found for some Iberian (GI) and eastern Mediterranean and Balearic (ITS) *B. hybridum* groups (Table 1; Figs. 2, 3). Furthermore, the low mean ‘interspecific’ divergence rates shown by the *B. distachyon*-like and *B. stacei*-like sequences of *B. hybridum* with respect to those of the two progenitors for the three studied loci (Table 2) indicate that the two genomes of the hybrid have kept the same or similar signatures as those of the ancestral genomes, supporting the recent origin of *B. hybridum* in the Pleistocene (cf. [11]). Additionally, the low mean ‘intraspecific’ divergence rates of the respective *B. distachyon*-like and *B. stacei*-like sequences of *B. hybridum* (Table 2), which are similar to the parental ones, suggests that the original genomes have remained largely intact and that the time elapsed since the hybridizations took part was a brief one. Nonetheless, the detection of some interspecific ITS and GI recombinant sequences in *B. hybridum* (Table 1; Figs. 2, 3) points towards the occurrence of frequent genomic rearrangements within the hybrid nucleus. This agrees with cytogenetic CCP evidence demonstrating the existence of structural rearrangements in the *B. hybridum* chromosomes with respect to the *B. distachyon* and *B. stacei* ones [16].

The recurrent formation of allopolyploid plant species has been largely documented in the literature [47], [48] and references therein). Their predominance over their parental diploid progenitors has been explained as the result of their higher fitness or their higher capability to colonize new habitats and new lands [49], [50]. The wide distribution of *B. hybridum*, which exceeds those of *B. distachyon* and *B. stacei* in their native Mediterranean region, as the only known species of the complex to have colonized other continents [9], , could be a consequence of its more genetically diverse hybrid genome and the likely recurrent origin of new hybrid variants. This could have resulted in fit and well adapted individuals that have displaced the parental species from their habitats and/or have invaded new niches [50]. Current studies are under way to investigate the recurrent origins of *B. hybridum* through time (López-Alvarez & Catalán, unpublished results).

### Future Perspectives of the Barcoding Method for Other *Brachypodium* Taxa

The almost exclusively self-fertile breeding system of the cleistogamous *B. distachyon* [8] and of *B. stacei* (L. Mur, pers. comm.) resulted in highly homozygous genomes of the two diploid parental species that contributed to the heterozygous allotetraploid *B. hybridum* genome [41]. In a recent assessment of genetic distances between different parent-pairs of hybrid plants, Paun and co-workers [51] concluded that parental species of allopolyploids were genetically more divergent that those of homoploid hybrids. Within *Brachypodium*, the differences in the inter- vs. intraspecific divergence values between the *B. stacei* and *B. distachyon* sequences were significant (Table 2). Catalán and co-workers [11] also found significant differences in the evolutionary rates of the *B. stacei* and *B. distachyon* ITS sequences, the former being significantly higher than the later. The salient features of the two distinct genomes were demonstrated through incompatible cross-GISH hybridizations [13], [14]. Their genomic divergences could have triggered the allopolyploidization process that resulted in the *B. hybridum* populations, and the long isolation of the two parental taxa has facilitated the detection of the proposed *trn*LF - ITS - GI barcoding method to distinguish the parents and the hybrid.

The usefulness of our DNA barcoding approach at the generic level could however be less successful among recently evolved taxa, like the core-perennial group of *Brachypodium* species, due to their close relationships [11], [52]. No significant differences in plastid *trn*LF and nuclear ITS sequences were detected between pairs of long rhizomatous *Brachypodium* species, nor between them and *B. distachy*on [11]. They were found, however, between the ancestral short-rhizomatous *B. mexicanum* and annual *B. stacei* taxa. Widespread geographical sampling would be required to test the utility of the *trn*LF and ITS barcodes within *Brachypodium* as a whole. Regarding GI, all the six analysed *Brachypodium* species [17] showed different sequences and copies, with copy numbers related to their ploidy levels. The apparently more-promising GI barcode should also be evaluated within a wide geographical and taxonomical sample of *Brachypodium* representatives. *Brachypodium* has been proposed as a model plant genus for temperate grasses [15], based on the overall small genome size of its members, their compact genomes and an extensive reticulate evolutionary and polyploid history [16]. Diverse stable species (e. g. *B. phoenicoides*, 2n = 4x = 28) and cytotypes (e. g. *B. pinnatum* 2n = 4x = 28) are of hybrid origin [16], [17] and most of the polyploids (e. g. *B. mexicanum*, *B. retusum*) are of suspected hybrid origin. Further research is currently under way to find a universal barcoding system for *Brachypodium*.

## Materials and Methods

### Sampling

A total of 210 samples (56 of *B. distachyon*, 43 of *B. stacei* and 111 of *B. hybridum*) were included in the study (Table 1). Those samples corresponded to inbred lines generated at CRF-INIA, INRA, USDA, and Aberystwyth (ABR), Alcalá de Henares (UAH), Jaén (UJA), Politécnica de Madrid (UPM), Tel-Aviv (TAU) and Zaragoza (Unizar) Universities and to new germplasm accessions collected over their entire native distribution areas in the Mediterranean region (Fig. 1). The identity of most of the samples was tested through DAPI-staining chromosome counting of the studied materials, which has proved to be the most accurate cytogenetic method to differentiate the three taxa. This was coupled with other identifications based on flow cytometry measurements (genome size) and anatomical stomata-leaf guard cell length measurements which separated, respectively, *B. hybridum* from the diploids, and all the three species [11]. We used this information as an *a priori* method to validate the resolution power of the proposed barcodes for the discrimination of the three species amplified with highly conserved primers.

### DNA Barcode Sequences of Plastid and Nuclear Genes

Three loci were tested as potential tools for effective DNA barcoding of *B. distachyon*, *B. stacei* and *B. hybridum*, the maternally inherited plastid *trn*LF region (*trn*L(UAA) intron - *trn*L(UAA) exon - *trn*L(UAA)/*trn*F(GAA) spacer) and the biparentally inherited nuclear multicopy ribosomal internal transcribed spacer ITS region (ITS1-5.8S-ITS2) and single-copy *GIGANTEA* (GI) gene. Total DNA was extracted from dried leaf tissue using a modified CTAB method of Doyle and Doyle [53]. The plastid *trn*LF region was amplified and sequenced from direct PCR products using the universal primers ‘c’ forward (5′-CGAAATCGGTAGACGCTACG-3′) and ‘f’ reverse (5′-ATTTGAACTGGTGACACGAG-3′) of Taberlet and co-workers [54]. PCR products were purified using ExoSAP-ITTM (USB Corporation, Cleveland, OH) and sequenced in both directions by cycle-sequencing using the Big-Dye version 3 chemistry (Perkin-Elmer), with a Prism 3100 Genetic Analyzer (ABI). The nuclear multicopy ITS region was amplified using primers ITSL forward (5′-TCGTAACAAGGTTTCCGTAGGTG-3′) and ITS4 reverse (5′-TCCTCCGCTTATTGATATGC-3′) optimized for grasses [55]. A 665 bp portion of the nuclear single-copy GI gene was amplified with primers GIGIE1F (5′-TATGTCWGYNTCAAATGGGAAGTGG-3′) and HGIE5R (5′-AACTTTRAAGATTGGCCTRTTGTRGTGA-3′) designed for *Brachypodium* [56]. Due to the, respectively, plausible and known existence of multiple ITS and GI copies in the *B. hybridum* samples, and the potential existence of more than one ITS copy in the *B. distachyon* and *B. stacei* samples, all GI and most ITS amplified products were cloned and sequenced, aiming to detect their potential intraindividual copy number variation. Sixty one amplified ITS (24) and GI (37) fragments were cloned separately into a pGEM®-T Vector System I cloning vector (Promega, USA) following the manufacturers’ instructions. These were transformed into *Escherichia coli* JM109 competent cells. Five colonies of each individual sample were randomly picked for a first ITS and GI screen, and each clone was sequenced using M13 forward (5′-GTTTTCCCAGTCACGAC-3′) and reverse (5′-CAGGAAACAGCTATGAC-3′ ) primers following the same procedure as for direct-PCR products. In some ambiguous cases, up to 10–16 (58) clones per individual sample had to be sequenced to detect the copy number variation (see Results). The characteristics of the amplification conditions for each barcoding gene are indicated in Supplemental methods (see Methods S1).

### Data Analyses

#### Data alignment

The independent *trn*LF, ITS and GI sequence data matrices were aligned using Geneious 4.7 (Biomatters, Auckland, New Zealand) and MacClade v4.08 [57]. The alignments were edited manually to remove adapters, PCR primers and bases that had been added during the ligation process and to optimize the final alignment. A single consensus sequence per individual sample was obtained from direct-PCR sequencing of the *trn*LF region (and in some cases of the ITS region) and several sequences per individual sample were obtained from the cloned ITS and GI regions (see Results).

#### Genetic distance analysis

Because the fundamental requirement of a suitable barcoding method is to attain a level of interspecific polymorphism high enough to allow the monophyletic grouping of individuals from the same species and the recovery of distinct clusters at the interspecific level [21], inter- and intraspecific genetic distances among and within *B. distachyon*, *B. stacei* and *B. hybridum* (*B. distachyon*- and *B. stacei*-like) sequences were calculated using the Kimura’s 2-parameter (K2P) model implemented in MEGA4 [58] for each DNA barcoding locus. The K2P method has been reported as a fast and accurate method to examine relationships among species and to assign unidentified samples to known species [59]. Due to the distinct nature and inheritance of each molecule, we analysed each locus separately rather than combining sequences. We also generated the respective Neighbor-Joining (NJ) trees based on the K2P sequence divergences estimated. A species was discriminated when more than 50% of the sampled individuals fell in the same monophyletic group in the NJ tree. This relatively low threshold has been chosen to reflect the minimum probability for which a correct identification would be more likely than a wrong identification [21].

#### Haplotype network analysis

The number of haplotypes of each separate locus was obtained from statistical parsimony analysis of the complete and unambiguous *trn*LF, ITS and GI aligned data matrices using TCS 1.21 [60]. The respective haplotype networks were constructed with this software imposing a 95% connection limit for up to 30 steps and treating the gaps as a 5^th^ character state. The clustering of similar haplotypes in groups and their divergence, based on the large number of mutational steps, were used as additional evidence supporting the barcoding method.

#### Phylogenetic analysis

Independent analyses were conducted on each separate data matrix that included, respectively, all the newly sequenced *B. distachyon*, *B. stacei* and *B. hybridum* samples, other representatives of the close *Brachypodium* perennials and other more distantly related Triticeae (*Hordeum, Secale*) outgroups that were used to root the trees (Table 1). The DNA sequences of the perennial *Brachypodium* taxa and of the outgroups corresponded to those analysed in the study of Catalán and co-workers [11]. Bayesian phylogenetic analyses were performed using the program MrBayes 3.1.2 [61]. The best nucleotide substitution model (GTR+ Г) was previously selected using the hierarchical likelihood ratio test and the Akaike criterion implemented in MrModeltest 2.3 [62]. The Bayesian Inference search was computed imposing the nst = 6 and rates = gamma parameters to the nucleotide sequence partition and leaving the program to estimate the remaining parameters. A total of 3750 posterior probability Bayesian trees were saved for each separate data matrix after performing two runs, each with 5000000 generations and four chains, sampling trees every 1000 generations, and a burn-in option of 1250 trees per run once stability in the likelihood values was attained. A Bayesian halfcompat consensus tree of all saved trees was computed for each separate data set; the posterior probability values of the branches of each consensus tree were used as a measure of their nodal support.

## Supporting Information

Figure S1 **Neighbor-Joining trees of the** ***Brachypodium distachyon*** **s. l. taxa (** ***B. distachyon*** **(blue),** ***B. stacei*** **(red) and** ***B. hybridum*** **(purple) based on pairwise K2P distances of DNA sequences.** a) *trn*LF tree; b) ITS tree; c) GI tree. Potential recombinant parental sequences of *B. hybridum* (BdisBsta, see S2) are indicated in green. ‘i’ and ‘am’ indicate, respectively, incomplete and ambiguous sequences. Numbers below branches correspond to bootstrap support (BS) values above 50%.
(TIF)

Table S1 Genbank accession numbers of the *Brachypodium distachyon*, *B. stacei* and *B. hybridum trn*LF, ITS and GI sequences. Newly deposited accession numbers are indicated in bold.
(DOCX)

Table S2 *List of Brachypodium distachyon*, *B. stacei* and *B. hybridum* haplotypes obtained from statistical parsimony analysis (TCS), treating the gaps as a 5^th^ character state, for the complete sets of *trn*LF, ITS and GI sequences (Table 1). The haplotypes have been classified as *B. distachyon*-type (Bdis) and *B. stacei*-type (Bsta) for each separate locus. Potential interspecific *B. distachyon* - *B. stacei* ITS and GI recombinant sequences found in *B. hybridum* are indicated as BdisBsta.
(DOCX)

Table S3 Characteristics of the studied *Brachypodium distachyon* s. l. complex (*B. distachyon, B. stacei, B. hybridum*) *trn*LF, ITS and GI sequences.
(DOCX)

Methods S1 **Supplemental methods.**
(DOCX)
