# Supplementary figures and images for: A DNA Barcoding Method to Discriminate between the Model Plant Brachypodium distachyon and Its Close Relatives B. stacei and B. hybridum (Poaceae)
Source: PLoS One. 2012 Dec 11;7(12):e51058. doi: 10.1371/journal.pone.0051058 (PMC3519806; doi:10.1371/journal.pone.0051058)

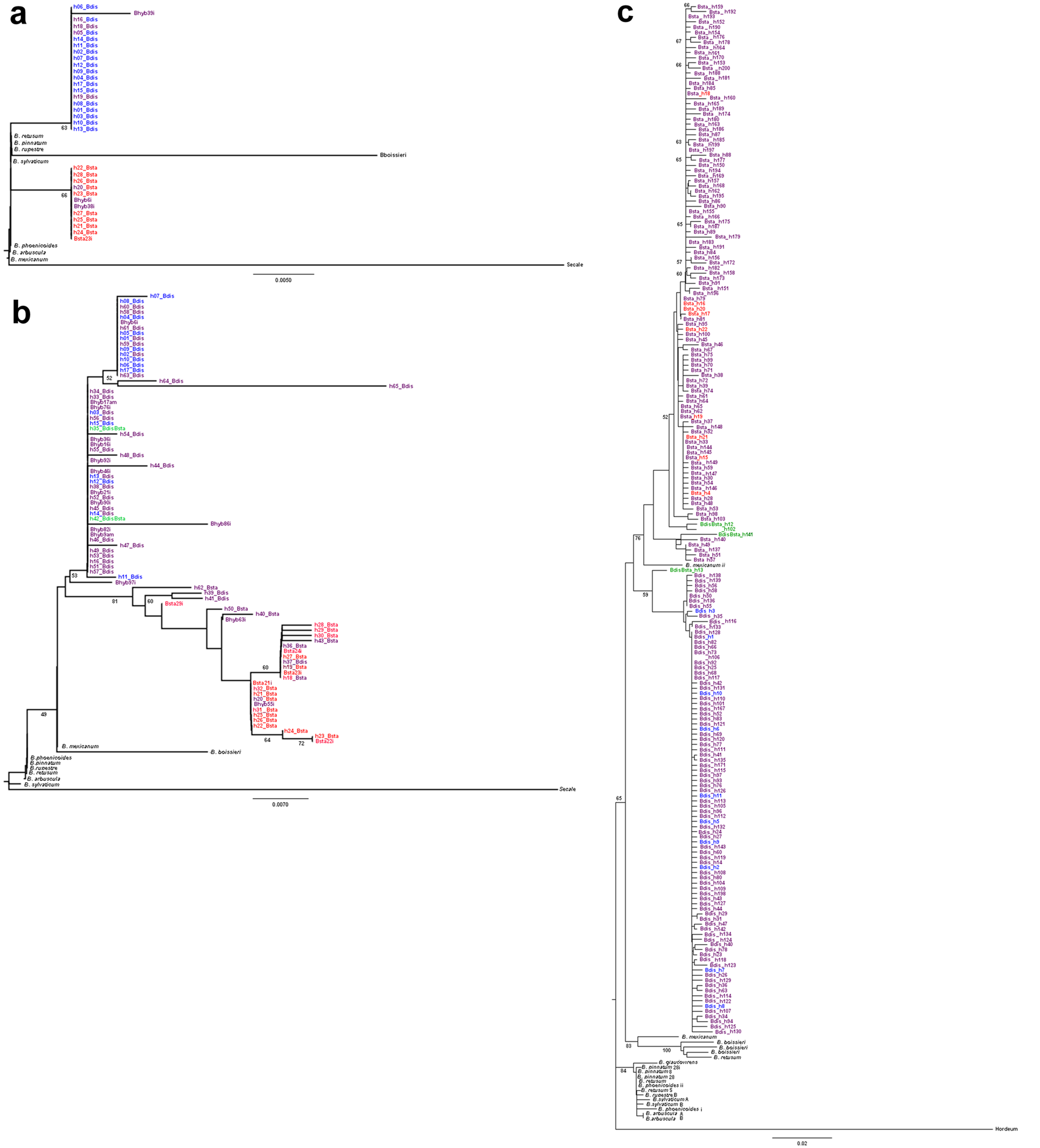

Supplement: Figure S1 — Neighbor-Joining trees of the Brachypodium distachyon s. l. taxa ( B. distachyon (blue), B. stacei (red) and B. hybridum (purple) based on pairwise K2P distances of DNA sequences. a) trnLF tree; b) ITS tree; c) GI tree. Potential recombinant parental sequences of B. hybridum (BdisBsta, see S2) are indicated in green. ‘i’ and ‘am’ indicate, respectively, incomplete and ambiguous sequences. Numbers below branches correspond to bootstrap support (BS) values above 50%. (TIF) [file pone.0051058.s001.tif]
