# Supplementary material for: A DNA Barcoding Method to Discriminate between the Model Plant Brachypodium distachyon and Its Close Relatives B. stacei and B. hybridum (Poaceae)
Source: PLoS One. 2012 Dec 11;7(12):e51058. doi: 10.1371/journal.pone.0051058 (PMC3519806; doi:10.1371/journal.pone.0051058)
Supplement: Table S1 — Genbank accession numbers of the Brachypodium distachyon, B. stacei and B. hybridum trnLF, ITS and GI sequences. Newly deposited accession numbers are indicated in bold. (DOCX) [file pone.0051058.s002.docx]

Table S1. Genbank accession numbers of the *Brachypodium distachyon*, *B. stacei* and *B. hybridum* *trn*LF, ITS and GI sequences. Newly deposited accession numbers are indicated in bold.

| Taxon | Code | trnLF | ITS | GI |
| --- | --- | --- | --- | --- |
| *B.distachyon* | Bdis1 | **JX665833** | **JX665532** |  |
| *B.distachyon* | Bdis2 | **JX665834** | **JX665533** |  |
| *B.distachyon* | Bdis3 | **JX665835** | **JX665534** |  |
| *B.distachyon* | Bdis4 | **JX665836** | **JX665535** |  |
| *B.distachyon* | Bdis5 | **JX665837** | **JX665536** |  |
| *B.distachyon* | Bdis6 | **JX665838** | **JX665537** | **JX666039** |
| *B.distachyon* | Bdis7 | **JX665839** | **JX665538** |  |
| *B.distachyon* | Bdis8 | **JX665840** | **JX665539** |  |
| *B.distachyon* | Bdis9 | **JX665841** | **JX665540** |  |
| *B.distachyon* | Bdis10 | **JX665842** |  |  |
| *B.distachyon* | Bdis11 | **JX665843** | **JX665541** |  |
| *B.distachyon* | Bdis12 | **JX665844** | **JX665542** |  |
| *B.distachyon* | Bdis13 | **JX665845** | **JX665543** |  |
| *B.distachyon* | Bdis14 | **JX665846** | **JX665544** | **JX666040** |
| *B.distachyon* | Bdis15 | **JX665847** | **JX665545** |  |
| *B.distachyon* | Bdis16 | **JX665848** | **JX665546** |  |
| *B.distachyon* | Bdis17 | JN187656 | JN187608 | **JX666041** |
| *B.distachyon* | Bdis18 |  | **JX665548- JX665550** | HQ890972 |
| *B.distachyon* | Bdis19 | JN187661 | JN187613 |  |
| *B.distachyon* | Bdis20 | JN187663 |  |  |
| *B.distachyon* | Bdis21 | JN187665 | JN187615**; JX665553-JX665557** |  |
| *B.distachyon* | Bdis22 | JN187616 | JN187593**; JX665559-JX665563** |  |
| *B.distachyon* | Bdis23 | **JX665854** | **JX665564-JX665569** | **JX666043-JX666047** |
| *B.distachyon* | Bdis24 | **JX665855** | **JX665570** |  |
| *B.distachyon* | Bdis25 | **JX665856** | **JX665571** |  |
| *B.distachyon* | Bdis26 | **JX665857** | **JX665572** |  |
| *B.distachyon* | Bdis27 | **JX665858** | **JX665573** | **JX666048-JX666052** |
| *B.distachyon* | Bdis28 | **JX665859** | **JX665574** |  |
| *B.distachyon* | Bdis29 | **JX665860** | **JX665575** |  |
| *B.distachyon* | Bdis30 | **JX665861** | **JX665576** |  |
| *B.distachyon* | Bdis31 | **JX665862** |  |  |
| *B.distachyon* | Bdis32 | **JX665863** |  |  |
| *B.distachyon* | Bdis33 | **JX665864** |  | **JX666053** |
| *B.distachyon* | Bdis34 | **JX665865** |  |  |
| *B.distachyon* | Bdis35 | **JX665866** |  |  |
| *B.distachyon* | Bdis36 | **JX665867-JX665869** | **JX665577-JX665582** |  |
| *B.distachyon* | Bdis37 | **JX665870** |  | **JX666054-JX666058** |
| *B.distachyon* | Bdis38 | **JX665871** | **JX665583** | **JX666059** |
| *B.distachyon* | Bdis39 | **JX665872** | **JX665584** | **JX666060** |
| *B.distachyon* | Bdis40 | **JX665873** | **JX665585** |  |
| *B.distachyon* | Bdis41 | **JX665874** | **JX665586** |  |
| *B.distachyon* | Bdis42 | **JX665875** | **JX665587** |  |
| *B.distachyon* | Bdis43 | **JX665876** | **JX665588** |  |
| *B.distachyon* | Bdis44 | **JX665877** | **JX665589** | **JX666061** |
| *B.distachyon* | Bdis45 | **JX665878** | **JX665590** |  |
| *B.distachyon* | Bdis46 | **JX665879** | **JX665591** |  |
| *B.distachyon* | Bdis47 | **JX665880** | **JX665592** |  |
| *B.distachyon* | Bdis48 | **JX665881** | **JX665593** | **JX666062** |
| *B.distachyon* | Bdis49 | **JX665882** | **JX665594** |  |
| *B.distachyon* | Bdis50 | **JX665883** | **JX665595** |  |
| *B.distachyon* | Bdis51 | **JX665884** | **JX665596** |  |
| *B.distachyon* | Bdis52 | **JX665885** | **JX665597** |  |
| *B.distachyon* | Bdis53 | **JX665886** | **JX665598** |  |
| *B.distachyon* | Bdis54 | **JX665888** |  |  |
| *B.distachyon* | Bdis55 | **JX665889** | **JX665600** | **JX666066** |
| *B.distachyon* | Bdis56 | **JX665890** | **JX665601** |  |
| *B.stacei* | Bsta1 | JN187659 | JN187611; **JX665763-JX665766** | HQ890969 |
| *B.stacei* | Bsta2 | **JX666000** | **JX665767** |  |
| *B.stacei* | Bsta3 | **JX666001** | **JX665768-JX665773** | **JX666224- JX666228** |
| *B.stacei* | Bsta4 | **JX666002** | **JX665774-JX665779** | **JX666229- JX666233** |
| *B.stacei* | Bsta5 | **JX666003** | **JX665780-JX665785** |  |
| *B.stacei* | Bsta6 | **JX666004** | **JX665786** |  |
| *B.stacei* | Bsta7 | **JX666005** | **JX665787-JX665792** |  |
| *B.stacei* | Bsta8 | **JX666006** | **JX665793** |  |
| *B.stacei* | Bsta9 | **JX666007** | **JX665794** | **JX666234** |
| *B.stacei* | Bsta10 | **JX666008** | **JX665795** |  |
| *B.stacei* | Bsta11 | **JX666009** | **JX665796** |  |
| *B.stacei* | Bsta12 | **JX666010** | **JX665797** |  |
| *B.stacei* | Bsta13 | **JX666011** | **JX665798** |  |
| *B.stacei* | Bsta14 | **JX666012** | **JX665799** |  |
| *B.stacei* | Bsta15 | **JX666013** | **JX665800** |  |
| *B.stacei* | Bsta16 | **JX666014** | **JX665801** |  |
| *B.stacei* | Bsta17 | **JX666015** | **JX665802** |  |
| *B.stacei* | Bsta18 | **JX666016** | **JX665803** |  |
| *B.stacei* | Bsta19 | **JX666017** | **JX665804** |  |
| *B.stacei* | Bsta20 | **JX666018** | **JX665805** | **JX666235** |
| *B.stacei* | Bsta21 | **JX666019** | **JX665806** |  |
| *B.stacei* | Bsta22 | **JX666020** | **JX665807** | **JX666236** |
| *B.stacei* | Bsta23 | **JX666021** | **JX665808** |  |
| *B.stacei* | Bsta24 | **JX666022** | **JX665809-JX665814** |  |
| *B.stacei* | Bsta25 | **JX666023** | **JX665815** |  |
| *B.stacei* | Bsta26 | **JX666024** | **JX665816** | **JX666237** |
| *B.stacei* | Bsta27 | **JX666025** | **JX665817** | **JX666238** |
| *B.stacei* | Bsta28 | **JX666026** | **JX665818** |  |
| *B.stacei* | Bsta29 |  | **JX665819** |  |
| *B.stacei* | Bsta30 | **JX666027** | **JX665820** |  |
| *B.stacei* | Bsta31 |  | **JX665821** |  |
| *B.stacei* | Bsta32 | **JX666028** | **JX665822** |  |
| *B.stacei* | Bsta33 | **JX666029** | **JX665823** |  |
| *B.stacei* | Bsta34 |  | **JX665824** |  |
| *B.stacei* | Bsta35 | **JX666030** | **JX665825** |  |
| *B.stacei* | Bsta36 | **JX666031** | **JX665826** | **JX666239** |
| *B.stacei* | Bsta37 | **JX666032** | **JX665827** |  |
| *B.stacei* | Bsta38 | **JX666033** | **JX665828** | **JX666240** |
| *B.stacei* | Bsta39 | **JX666034** |  |  |
| *B.stacei* | Bsta40 | **JX666035** | **JX665829** |  |
| *B.stacei* | Bsta41 | **JX666036** | **JX665830** |  |
| *B.stacei* | Bsta42 | **JX666037** | **JX665831** | **JX666241** |
| *B.stacei* | Bsta43 | **JX666038** | **JX665832** |  |
| *B.hybridum* | Bhyb1 | **JX665891** | **JX665602** | **JX666067-JX666071** |
| *B.hybridum* | Bhyb2 | **JX665892** | **JX665603** | **JX666072-JX666081; JX967124-JX967137; JX967186-JX967220** |
| *B.hybridum* | Bhyb3 | **JX665893** |  |  |
| *B.hybridum* | Bhyb4 | **JX665894** | **JX665604** |  |
| *B.hybridum* | Bhyb5 | **JX665895** | **JX665605** |  |
| *B.hybridum* | Bhyb6 | **JX665896** | **JX665606** |  |
| *B.hybridum* | Bhyb7 | **JX665897** |  | **JX666082-JX666086** |
| *B.hybridum* | Bhyb8 | **JX665898** | **JX665607-JX665612** | **JX666087-JX666090** |
| *B.hybridum* | Bhyb9 | JN187662 | **JX665613-JX665618** |  |
| *B.hybridum* | Bhyb10 | JN187664 | JN187614; **JX665620-JX665623** | **JX666091-JX666095** |
| *B.hybridum* | Bhyb11 | JN187658 | JN187610; **JX665625-JX665627** | HQ890970/ HQ890973 |
| *B.hybridum* | Bhyb12 | JN187657 | JN187609 |  |
| *B.hybridum* | Bhyb13 | JN187660 | JN187612**; JX665630-JX665632** | **JX666098-JX666104** |
| *B.hybridum* | Bhyb14 | JN187667 | **JX665633- JX665638** |  |
| *B.hybridum* | Bhyb15 | JN187668 | JN187617**; JX665640-JX665644** |  |
| *B.hybridum* | Bhyb16 | **JX665906** | **JX665645** |  |
| *B.hybridum* | Bhyb17 | **JX665907** | **JX665646** |  |
| *B.hybridum* | Bhyb18 | **JX665908** | **JX665647-JX665652** | **JX666105- JX666109** |
| *B.hybridum* | Bhyb19 | **JX665909** | **JX665653-JX665657** | **JX666110- JX666114** |
| *B.hybridum* | Bhyb20 | **JX665910** | **JX665658** |  |
| *B.hybridum* | Bhyb21 | **JX665911** | **JX665659** |  |
| *B.hybridum* | Bhyb22 | **JX665912** | **JX665660-JX665665** |  |
| *B.hybridum* | Bhyb23 | **JX665913** | **JX665666-JX665670** |  |
| *B.hybridum* | Bhyb24 | **JX665914** | **JX665671** |  |
| *B.hybridum* | Bhyb25 | **JX665915** | **JX665672** |  |
| *B.hybridum* | Bhyb26 | **JX665916** | **JX665673** | **JX666115- JX666119** |
| *B.hybridum* | Bhyb27 | **JX665917** | **JX665674** |  |
| *B.hybridum* | Bhyb28 | **JX665918** | **JX665675** | **JX666120- JX666124** |
| *B.hybridum* | Bhyb29 | **JX665919** | **JX665676** |  |
| *B.hybridum* | Bhyb30 | **JX665920** | **JX665677** | **JX666125- JX666129** |
| *B.hybridum* | Bhyb31 | **JX665921** | **JX665678** |  |
| *B.hybridum* | Bhyb32 | **JX665922** | **JX665679** |  |
| *B.hybridum* | Bhyb33 | **JX665923** | **JX665680** |  |
| *B.hybridum* | Bhyb34 | **JX665924** | **JX665681** | **JX666130- JX666133; JX967170-JX967185** |
| *B.hybridum* | Bhyb35 | **JX665925** | **JX665682** | **JX666134- JX666138; JX967137-JX967153** |
| *B.hybridum* | Bhyb36 | **JX665926** | **JX665683** |  |
| *B.hybridum* | Bhyb37 | **JX665927** | **JX665684** |  |
| *B.hybridum* | Bhyb38 | **JX665928** | **JX665685-JX665690** |  |
| *B.hybridum* | Bhyb39 | **JX665929** |  |  |
| *B.hybridum* | Bhyb40 | **JX665930** | **JX665691** | **JX666139- JX666143** |
| *B.hybridum* | Bhyb41 | **JX665931** | **JX665692** | **JX666144- JX666148** |
| *B.hybridum* | Bhyb42 | **JX665932** | **JX665693** |  |
| *B.hybridum* | Bhyb43 | **JX665933** | **JX665694** |  |
| *B.hybridum* | Bhyb44 | **JX665934** | **JX665695** |  |
| *B.hybridum* | Bhyb45 | **JX665935** | **JX665696** |  |
| *B.hybridum* | Bhyb46 | **JX665936** | **JX665697** | **JX666149- JX666153** |
| *B.hybridum* | Bhyb47 | **JX665937** | **JX665698** | **JX666154- JX666157** |
| *B.hybridum* | Bhyb48 | **JX665938** | **JX665699** |  |
| *B.hybridum* | Bhyb49 | **JX665939** | **JX665700** |  |
| *B.hybridum* | Bhyb50 | **JX665940** | **JX665701** | **JX666158- JX666167** |
| *B.hybridum* | Bhyb51 | **JX665941** | **JX665702** | **JX666168- JX666172** |
| *B.hybridum* | Bhyb52 | **JX665942** | **JX665703** |  |
| *B.hybridum* | Bhyb53 | **JX665943** | **JX665704** |  |
| *B.hybridum* | Bhyb54 | **JX665887** | **JX665599** | **JX666063-JX666065** |
| *B.hybridum* | Bhyb55 | **JX665944** | **JX665705** |  |
| *B.hybridum* | Bhyb56 | **JX665945** | **JX665706** |  |
| *B.hybridum* | Bhyb57 | **JX665946** | **JX665707** |  |
| *B.hybridum* | Bhyb58 | **JX665947** | **JX665708** |  |
| *B.hybridum* | Bhyb59 | **JX665948** | **JX665709** |  |
| *B.hybridum* | Bhyb60 | **JX665949** | **JX665710** |  |
| *B.hybridum* | Bhyb61 | **JX665950** | **JX665711** |  |
| *B.hybridum* | Bhyb62 | **JX665951** | **JX665712** | **JX666173- JX666177** |
| *B.hybridum* | Bhyb63 |  | **JX665713** | **JX666178- JX666182** |
| *B.hybridum* | Bhyb64 | **JX665952** | **JX665714** |  |
| *B.hybridum* | Bhyb65 | **JX665953** | **JX665715** | **JX666183- JX666187** |
| *B.hybridum* | Bhyb66 | **JX665954** | **JX665716** |  |
| *B.hybridum* | Bhyb67 | **JX665955** | **JX665717** |  |
| *B.hybridum* | Bhyb68 | **JX665956** | **JX665718** |  |
| *B.hybridum* | Bhyb69 | **JX665957** | **JX665719** | **JX666188- JX666197; JX967154-JX967169; JX967221-JX967262** |
| *B.hybridum* | Bhyb70 | **JX665958** | **JX665720** |  |
| *B.hybridum* | Bhyb71 | **JX665959** | **JX665721** | **JX666198- JX666202** |
| *B.hybridum* | Bhyb72 | **JX665960** | **JX665722** |  |
| *B.hybridum* | Bhyb73 | **JX665961** | **JX665723** |  |
| *B.hybridum* | Bhyb74 | **JX665962** | **JX665724** |  |
| *B.hybridum* | Bhyb75 | **JX665963** | **JX665725** |  |
| *B.hybridum* | Bhyb76 | **JX665964** | **JX665726** |  |
| *B.hybridum* | Bhyb77 | **JX665965** | **JX665727** |  |
| *B.hybridum* | Bhyb78 | **JX665966** | **JX665728** |  |
| *B.hybridum* | Bhyb79 | **JX665967** | **JX665729** |  |
| *B.hybridum* | Bhyb80 | **JX665968** | **JX665730** | **JX666203- JX666207** |
| *B.hybridum* | Bhyb81 | **JX665969** | **JX665731** |  |
| *B.hybridum* | Bhyb82 | **JX665970** | **JX665732** |  |
| *B.hybridum* | Bhyb83 | **JX665971** | **JX665733** |  |
| *B.hybridum* | Bhyb84 | **JX665972** | **JX665734** | **JX666208- JX666210** |
| *B.hybridum* | Bhyb85 | **JX665973** | **JX665735** |  |
| *B.hybridum* | Bhyb86 | **JX665974** | **JX665736** | **JX666211- JX666215** |
| *B.hybridum* | Bhyb87 | **JX665975** | **JX665737** | **JX666216- JX666218** |
| *B.hybridum* | Bhyb88 | **JX665976** | **JX665738** |  |
| *B.hybridum* | Bhyb89 | **JX665977** | **JX665739** | **JX666219- JX666222** |
| *B.hybridum* | Bhyb90 | **JX665978** | **JX665740** |  |
| *B.hybridum* | Bhyb91 | **JX665979** | **JX665741** |  |
| *B.hybridum* | Bhyb92 | **JX665980** | **JX665742** |  |
| *B.hybridum* | Bhyb93 | **JX665981** | **JX665743** |  |
| *B.hybridum* | Bhyb94 | **JX665982** | **JX665744** |  |
| *B.hybridum* | Bhyb95 | **JX665983** | **JX665745** |  |
| *B.hybridum* | Bhyb96 | **JX665984** | **JX665746** |  |
| *B.hybridum* | Bhyb97 | **JX665985** | **JX665747** |  |
| *B.hybridum* | Bhyb98 | **JX665986** | **JX665748** |  |
| *B.hybridum* | Bhyb99 | **JX665987** | **JX665749** |  |
| *B.hybridum* | Bhyb100 | **JX665988** | **JX665750** |  |
| *B.hybridum* | Bhyb101 | **JX665989** | **JX665751** |  |
| *B.hybridum* | Bhyb102 | **JX665990** | **JX665752** |  |
| *B.hybridum* | Bhyb103 | **JX665991** | **JX665753** |  |
| *B.hybridum* | Bhyb104 | **JX665992** | **JX665754** |  |
| *B.hybridum* | Bhyb105 | **JX665993** | **JX665755** |  |
| *B.hybridum* | Bhyb106 | **JX665994** | **JX665756** |  |
| *B.hybridum* | Bhyb107 | **JX665995** | **JX665757** |  |
| *B.hybridum* | Bhyb108 | **JX665996** | **JX665758** |  |
| *B.hybridum* | Bhyb109 | **JX665997** | **JX665759** |  |
| *B.hybridum* | Bhyb110 | **JX665998** | **JX665760** |  |
| *B.hybridum* | Bhyb111 |  | **JX665761** |  |
