# Supplementary material for: A DNA Barcoding Method to Discriminate between the Model Plant Brachypodium distachyon and Its Close Relatives B. stacei and B. hybridum (Poaceae)
Source: PLoS One. 2012 Dec 11;7(12):e51058. doi: 10.1371/journal.pone.0051058 (PMC3519806; doi:10.1371/journal.pone.0051058)
Supplement: Table S2 — List of Brachypodium distachyon, B. stacei and B. hybridum haplotypes obtained from statistical parsimony analysis (TCS), treating the gaps as a 5th character state, for the complete sets of trnLF, ITS and GI sequences (Table 1). The haplotypes have been classified as B. distachyon-type (Bdis) and B. stacei-type (Bsta) for each separate locus. Potential interspecific B. distachyon - B. stacei ITS and GI recombinant sequences found in B. hybridum are indicated as BdisBsta. (DOCX) [file pone.0051058.s003.docx]

Table S2. *List of Brachypodium distachyon*, *B. stacei* and *B. hybridum* haplotypes obtained from statistical parsimony analysis (TCS), treating the gaps as a 5^th^ character state, for the complete sets of *trn*LF, ITS and GI sequences (Table 1). The haplotypes have been classified as *B. distachyon*-type (Bdis) and *B. stacei*-type (Bsta) for each separate locus. Potential interspecific *B. distachyon* - *B. stacei* ITS and GI recombinant sequences found in *B. hybridum* are indicated as BdisBsta.

*trn*LF locus

Number of haplotypes = 28

Number of sequences = 202

- h1-Bdis (1): Bdis1

- h2-Bdis (16): Bdis2 Bdis3 Bdis4 Bdis5 Bdis7 Bdis8 Bdis9 Bdis10 Bdis11 Bdis13 Bdis15 Bdis17 Bdis24 Bdis25 Bdis26 Bdis45

- h3-Bdis (3): Bdis6 Bdis12 Bdis14

- h4-Bdis (8): Bdis19 Bdis21 Bdis22 Bdis29 Bdis31 Bdis33 Bdis34 Bdis37

- h5-Bdis (12): Bdis20 Bdis39 Bdis40 Bdis41 Bdis42 Bdis43 Bdis44 Bdis46 Bdis47 Bdis48 Bdis49 Bhyb54

- h6-Bdis (2): Bdis23 Bdis56

- h7-Bdis (1): Bdis27

- h8-Bdis (1): Bdis28

- h9-Bdis (2): Bdis30 Bdis32

- h10-Bdis (1): Bdis35

- h11-Bdis (3): Bdis36_2 Bdis36_8 Bdis36_1

- h12-Bdis (1): Bdis38

- h13-Bdis (2): Bdis50 Bdis55

- h14-Bdis (1): Bdis51

- h15-Bdis (1): Bdis52

- h16-Bdis (2): Bdis53 Bhyb35

- h17-Bdis (1): Bdis54

- h18-Bdis (1): Bhyb26

- h19-Bdis (1): Bhyb30

- h20-Bsta (132): Bdis16 Bsta1 Bsta2 Bsta3 Bsta4 Bsta6 Bsta7 Bsta8 Bsta9 Bsta10 Bsta11 Bsta12 Bsta13 Bsta14 Bsta15 Bsta16 Bsta17 Bsta18 Bsta19 Bsta20 Bsta21 Bsta22 Bsta24 Bsta25 Bsta26 Bsta27 Bsta28 Bsta33 Bsta35 Bsta36 Bsta37 Bsta39 Bsta40 Bsta41 Bsta42 Bsta43 Bhyb1 Bhyb2 Bhyb3 Bhyb4 Bhyb5 Bhyb7 Bhyb8 Bhyb9

Bhyb10 Bhyb11 Bhyb12 Bhyb13 Bhyb15 Bhyb17 Bhyb18 Bhyb19 Bhyb20 Bhyb22 Bhyb23 Bhyb24 Bhyb25 Bhyb27 Bhyb28 Bhyb29 Bhyb31 Bhyb32 Bhyb33 Bhyb34 Bhyb36 Bhyb37 Bhyb40 Bhyb41 Bhyb42 Bhyb43 Bhyb44 Bhyb45 Bhyb46 Bhyb47 Bhyb48 Bhyb49 Bhyb50 Bhyb51 Bhyb52 Bhyb53 Bhyb55 Bhyb56 Bhyb57 Bhyb58 Bhyb60 Bhyb61 Bhyb62 Bhyb64 Bhyb66 Bhyb67 Bhyb68 Bhyb69 Bhyb70 Bhyb71 Bhyb72 Bhyb73 Bhyb74 Bhyb75 Bhyb76 Bhyb77 Bhyb78 Bhyb79 Bhyb80 Bhyb81 Bhyb82 Bhyb83 Bhyb84 Bhyb85 Bhyb86 Bhyb87 Bhyb88 Bhyb90 Bhyb91 Bhyb92 Bhyb93 Bhyb94 Bhyb95 Bhyb96 Bhyb97 Bhyb98 Bhyb99 Bhyb100 Bhyb101 Bhyb102 Bhyb103 Bhyb104 Bhyb105 Bhyb106 Bhyb107 Bhyb108 Bhyb109 Bhyb110

- h21-Bsta (2): Bsta5 Bsta38

- h22-Bsta (1): Bsta30

- h23-Bsta (1): Bsta32

- h24-Bsta (1): Bhyb14

- h25-Bsta (1): Bhyb16

- h26-Bsta (1): Bhyb21

- h27-Bsta (2): Bhyb59 Bhyb65

- h28-Bsta (1): Bhyb89

ITS locus

Number of haplotypes = 65

Number of sequences = 279

- h1-Bdis (31): Bdis1 Bdis5 Bdis11 Bdis15 Bdis16 Bdis17 Bdis23 Bdis23_1 Bdis23_2 Bdis23_3 Bdis23_4 Bdis28 Bdis29 Bdis44 Bdis45 Bdis46 Bhyb22 Bhyb22_2 Bhyb22_3 Bhyb22_4 Bhyb22_5 Bhyb29 Bhyb78 Bhyb93 Bhyb94 Bhyb95 Bhyb96 Bhyb102 Bhyb110 Bhyb111 Bhyb54

- h2-Bdis (32): Bdis2 Bdis3 Bdis6 Bdis7 Bdis8 Bdis12 Bdis13 Bdis14 Bdis19 Bdis21 Bdis21_1 Bdis21_3 Bdis21_5 Bdis22 Bdis22_1 Bdis22_2 Bdis22_3 Bdis22_4 Bdis22_5 Bdis24 Bdis25 Bdis26 Bhyb2 Bhyb14 Bhyb14_1 Bhyb14_2 Bhyb14_3 Bhyb14_4 Bhyb20 Bhyb27 Bhyb99 Bhyb106

- h3-Bdis (37): Bdis4 Bdis48 Bdis50 Bhyb4 Bhyb5 Bhyb8 Bhyb8_1 Bhyb8_3 Bhyb8_4 Bhyb9_1 Bhyb9_2 Bhyb9_4 Bhyb9_5 Bhyb13 Bhyb13_2 Bhyb13_3 Bhyb13_4 Bhyb24 Bhyb25 Bhyb32 Bhyb33 Bhyb34 Bhyb37 Bhyb38 Bhyb38_1 Bhyb38_2 Bhyb50 Bhyb57 Bhyb59 Bhyb62 Bhyb73 Bhyb74 Bhyb75 Bhyb77 Bhyb84 Bhyb88 Bhyb104

- h4-Bdis (1): Bdis9

- h5-Bdis (2): Bdis18_1 Bdis18_2

- h6-Bdis (1): Bdis18_3

- h7-Bdis (2): Bdis21_2 Bdis21_4

- h8-Bdis (1): Bdis23_5

- h9-Bdis (3): Bdis27 Bdis38 Bdis51

- h10-Bdis (1): Bdis30

- h11-Bdis (4): Bdis36_2 Bdis36_2_3 Bdis36_2_4 Bdis36_2_5

- h12-Bdis (1): Bdis36_8

- h13-Bdis (22): Bdis36_1 Bdis47 Bdis49 Bhyb10_2 Bhyb10_3 Bhyb10_5 Bhyb11_1 Bhyb11_3 Bhyb15 Bhyb15_2 Bhyb15_3 Bhyb15_4 Bhyb15_5 Bhyb19 Bhyb45 Bhyb48 Bhyb61 Bhyb70 Bhyb79 Bhyb80 Bhyb83 Bhyb91

- h14-Bdis (18): Bdis39 Bdis40 Bdis41 Bdis42 Bdis43 Bhyb12 Bhyb42 Bhyb43 Bhyb51 Bhyb52 Bhyb53 Bhyb58 Bhyb64 Bhyb66 Bhyb67 Bhyb68 Bhyb71 Bhyb85

- h15-Bdis (2): Bdis52 Bdis53

- h16-Bdis (1): Bdis55

- h17-Bdis (1): Bdis56

- h18-Bsta (19): Bsta1 Bsta1_1 Bsta1_2 Bsta1_4 Bsta25 Bsta27 Bsta28 Bsta31 Bsta34 Bhyb9_3 Bhyb14_5 Bhyb15_1 Bhyb19_4 Bhyb22_1 Bhyb23 Bhyb23_1 Bhyb23_3 Bhyb23_4 Bhyb28

- h19-Bsta (1): Bsta1_5

- h20-Bsta (25): Bsta2 Bsta6 Bsta7_1 Bsta7_3 Bsta7_4 Bsta7_5 Bsta11 Bsta12 Bsta13 Bsta14 Bsta15 Bsta16 Bsta17 Bsta18 Bsta19 Bsta20 Bsta30 Bsta35 Bhyb18_2 Bhyb18_4 Bhyb18_5 Bhyb38_5 Bhyb40 Bhyb41 Bhyb49

- h21-Bsta (6): Bsta3 Bsta3_1 Bsta3_2 Bsta3_3 Bsta3_4 Bsta3_5

- h22-Bsta (13): Bsta4 Bsta4_1 Bsta4_2 Bsta4_3 Bsta4_4 Bsta4_5 Bsta9 Bsta32 Bsta33 Bsta36 Bsta37 Bsta42 Bsta43

- h23-Bsta (5): Bsta5 Bsta5_1 Bsta5_3 Bsta5_4 Bsta5_5

- h24-Bsta (3): Bsta5_2 Bsta10 Bsta38

- h25-Bsta (1): Bsta7

- h26-Bsta (1): Bsta7_2

- h27-Bsta (1): Bsta24_1

- h28-Bsta (1): Bsta24_2

- h29-Bsta (2): Bsta24_3 Bsta24_5

- h30-Bsta (1): Bsta24_4

- h31-Bsta (1): Bsta26

- h32-Bsta (1): Bsta41

- h33-Bdis (1): Bhyb1

- h34-Bdis (1): Bhyb8_2

- h35-BdisBsta (1): Bhyb8_5

- h36-Bsta (1): Bhyb10

- h37-Bdis (1): Bhyb10_1

- h38-Bdis (1): Bhyb11

- h39-Bdis (1): Bhyb11_2

- h40-Bsta (1): Bhyb18

- h41-Bdis (1): Bhyb18_1

- h42-BdisBsta (1): Bhyb18_3

- h43-Bsta (3): Bhyb19_1 Bhyb19_2 Bhyb19_5

- h44-Bdis (1): Bhyb23_5

- h45-Bdis (2): Bhyb26 Bhyb30

- h46-Bdis (1): Bhyb31

- h47-Bdis (1): Bhyb35

- h48-Bdis (2): Bhyb38_3 Bhyb38_4

- h49-Bdis (2): Bhyb44 Bhyb69

- h50-Bsta (1): Bhyb47

- h51-Bdis (1): Bhyb56

- h52-Bdis (1): Bhyb60

- h53-Bdis (1): Bhyb65

- h54-Bdis (1): Bhyb72

- h55-Bdis (1): Bhyb81

- h56-Bdis (1): Bhyb87

- h57-Bdis (1): Bhyb89

- h58-Bdis (1): Bhyb98

- h59-Bdis (1): Bhyb100

- h60-Bdis (1): Bhyb101

- h61-Bdis (1): Bhyb103

- h62-Bsta (1): Bhyb105

- h63-Bdis (1): Bhyb107

- h64-Bdis (1): Bhyb108

- h65-Bdis (1): Bhyb109

GI locus

Number of haplotypes = 200

Number of sequences = 342

- h1-Bdis (66): Bdis6 Bdis14 Bdis17 Bdis18 Bdis23_5 Bdis23_4 Bdis23_3 Bdis23_2 Bdis27_5 Bdis27_4 Bdis27_2 Bdis27_1 Bdis32_1 Bdis32_4 Bdis33 Bdis37_2 Bdis39 Bdis44 Bdis48 Bdis55 Bhyb2_1 Bhyb2_3 Bhyb2_4 Bhyb2_6 Bhyb7_4 Bhyb8_1 Bhyb8_2 Bhyb11 Bhyb19_1 Bhyb19_2 Bhyb28_5 Bhyb40_3 Bhyb41_4 Bhyb41_5 Bhyb47_2 Bhyb50_9 Bhyb51_2 Bhyb63_1 Bhyb65_2 Bhyb65_4 Bhyb65_5 Bhyb87_1 Bhyb87_3 Bhyb89_2 Bhyb2_3a Bhyb2_5a Bhyb2_7a Bhyb2_8a Bhyb2_15a Bhyb2_16a Bhyb34_3a Bhyb34_10a Bhyb34_4a Bhyb34_6a Bhyb2_62a Bhyb2_56a Bhyb2_49a Bhyb2_57a Bhyb2_36a Bhyb2_41a Bhyb2_44a Bhyb2_51a Bhyb2_60a Bhyb2_17a Bhyb2_27a Bhyb2_24a

- h2-Bdis (1): Bdis23_1

- h3-Bdis (1): Bdis27_3

- h4-Bsta (1): Bdis32_2

- h5-Bdis (1): Bdis32_3

- h6-Bdis (1): Bdis32_5

- h7-Bdis (1): Bdis37_1

- h8-Bdis (1): Bdis37_3

- h9-Bdis (1): Bdis37_4

- h10-Bdis (1): Bdis37_5

- h11-Bdis (1): Bdis38

- h12-BdisBsta (1): Bhyb54_1

- h13-BdisBsta (1): Bhyb54_2

- h14-Bdis (1): Bhyb54_3

- h15-Bsta (15): Bsta1 Bsta22 Bsta36 Bsta38 Bhyb8_5 Bhyb11_a Bhyb19_4 Bhyb19_5 Bhyb28_1 Bhyb34_3 Bhyb34_5 Bhyb34_8a Bhyb34_11a Bhyb34_7a Bhyb34_12a

- h16-Bsta (9): Bsta3_5 Bsta3_4 Bsta3_3 Bsta3_2 Bsta3_1 Bsta4_5 Bsta4_4 Bsta4_2 Bsta42

- h17-Bsta (9): Bsta4_3

- h18-Bsta (9): Bsta4_1 Bhyb69_6 Bhyb69_10 Bhyb69_11a Bhyb69_51a Bhyb69_60a Bhyb69_18a Bhyb69_17a Bhyb69_33a

- h19-Bsta (9): Bsta9 Bhyb40_1 Bhyb46_3 Bhyb46_5 Bhyb47_5 Bhyb62_4 Bhyb62_2 Bhyb89_1 Bhyb89_4

- h20-Bsta (1): Bsta20

- h21-Bsta (1): Bsta26

- h22-Bsta (1): Bsta27

- h23-Bdis (1): Bhyb2_2

- h24-Bdis (2): Bhyb2_5 Bhyb2_7

- h25-Bdis (1): Bhyb2_10

- h26-Bdis (1): Bhyb2_8

- h27-Bdis (1): Bhyb2_9

- h28-Bsta (1): Bhyb7_1

- h29-Bdis (1): Bhyb7_2

- h30-Bsta (1): Bhyb7_3

- h31-Bdis (1): Bhyb7_5

- h32-Bsta (1): Bhyb8_3

- h33-Bsta Bhyb10_1

- h34-Bdis (1): Bhyb10_2

- h35-Bdis (1): Bhyb10_3

- h36-Bdis (1): Bhyb10_4

- h37-Bsta (1): Bhyb10_5

- h38-Bsta (1): Bhyb13_8

- h39-Bsta (10): Bhyb13_7 Bhyb50_1 Bhyb50_10 Bhyb50_2 Bhyb50_8 Bhyb50_7 Bhyb50_5 Bhyb51_3 Bhyb65_3 Bhyb86_1

- h40-Bdis (1): Bhyb13_10

- h41-Bdis (1): Bhyb13_1

- h42-Bdis (1): Bhyb13_2

- h43-Bdis (1): Bhyb13_3

- h44-Bdis (1): Bhyb13_5

- h45-Bsta (2): Bhyb18_1 Bhyb18_3

- h46-Bsta (1): Bhyb18_2

- h47-Bdis (2): Bhyb18_4 Bhyb18_5

- h48-Bsta (1): Bhyb19_3

- h49-Bsta (5): Bhyb26_1 Bhyb26_2 Bhyb26_3 Bhyb35_7a Bhyb35_9a

- h50-Bdis (14): Bhyb26_4 Bhyb35_1 Bhyb35_2 Bhyb35_3 Bhyb35_4 Bhyb35_5 Bhyb35_1a Bhyb35_3a Bhyb35_4a Bhyb35_6a Bhyb35_10a Bhyb35_12a Bhyb35_14a Bhyb35_16a

- h51-Bsta (1): Bhyb26_5

- h52-Bdis (1): Bhyb28_2

- h53-Bsta (1): Bhyb28_3

- h54-Bsta (1): Bhyb28_4

- h55-Bdis (1): Bhyb30_1

- h56-Bdis (2): Bhyb30_2 Bhyb30_5

- h57-Bsta (1): Bhyb30_3

- h58-Bdis (1): Bhyb30_4

- h59-Bsta (2): Bhyb34_1 Bhyb34_4

- h60-Bdis (1): Bhyb40_2

- h61-Bsta (1): Bhyb40_4

- h62-Bsta (1): Bhyb40_5

- h63-Bdis (1): Bhyb41_1

- h64-Bsta (2): Bhyb41_2 Bhyb41_3

- h65-Bsta (1): Bhyb46_1

- h66-Bdis (1): Bhyb46_2

- h67-Bsta (1): Bhyb46_4

- h68-Bdis (1): Bhyb47_3

- h69-Bdis (1): Bhyb47_4

- h70-Bsta (1): Bhyb50_3

- h71-Bsta (1): Bhyb50_4

- h72-Bsta (1): Bhyb50_6

- h73-Bdis (1): Bhyb51_1

- h74-Bsta (1): Bhyb51_4

- h75-Bsta (1): Bhyb51_5

- h76-Bdis (1): Bhyb62_5

- h77-Bdis (1): Bhyb62_3

- h78-Bdis (1): Bhyb62_1

- h79-Bsta (1): Bhyb63_2

- h80-Bdis (1): Bhyb63_3

- h81-Bsta (1): Bhyb63_4

- h82-Bdis (1): Bhyb63_5

- h83-Bdis (1): Bhyb65_1

- h84-Bsta (1): Bhyb69_9

- h85-Bsta (1): Bhyb69_8

- h86-Bsta (1): Bhyb69_7

- h87-Bsta (1): Bhyb69_1

- h88-Bsta (1): Bhyb69_2

- h89-Bsta (1): Bhyb69_3

- h90-Bsta (1): Bhyb69_4

- h91-Bsta (1): Bhyb69_5

- h92-Bdis (1): Bhyb71_1

- h93-Bdis (1): Bhyb71_2

- h94-Bdis (1): Bhyb71_3

- h95-Bsta (1): Bhyb71_4

- h96-Bdis (1): Bhyb71_5

- h97-Bdis (4): Bhyb80_1 Bhyb80_3 Bhyb80_4 Bhyb80_5

- h98-Bsta (1): Bhyb80_2

- h99-Bsta (1): Bhyb84_1

- h100-Bsta (1): Bhyb84_2

- h101-Bdis (1): Bhyb84_3

- h102-BdisBsta (4): Bhyb86_2 Bhyb86_3 Bhyb86_4 Bhyb86_5

- h103-Bsta (1): Bhyb87_2

- h104-Bdis (1): Bhyb89_3

-h105-Bdis (1):Bhyb2_1a

-h106-Bdis (1):Bhyb2_2a

-h107-Bdis (1):Bhyb2_6a

-h108-Bdis (1):Bhyb2_9a

-h109-Bdis (1):Bhyb2_11a

-h110-Bdis (1):Bhyb2_12a

-h111-Bdis (1):Bhyb2_13a

-h112-Bdis (1):Bhyb2_14a

-h113-Bdis (1):Bhyb2_19a

-h114-Bdis (1):Bhyb2_20a

-h115-Bdis (1):Bhyb2_22a

-h116-Bdis (1):Bhyb2_23a

-h117-Bdis (1):Bhyb2_25a

-h118-Bdis (1):Bhyb2_26a

-h119-Bdis (1):Bhyb2_31a

-h120-Bdis (1):Bhyb2_32a

-h121-Bdis (1):Bhyb2_33a

-h122-Bdis (1):Bhyb2_34a

-h123-Bdis (1):Bhyb2_35a

-h124-Bdis (1):Bhyb2_37a

-h125-Bdis (1):Bhyb2_39a

-h126-Bdis (1):Bhyb2_40a

-h127-Bdis (1):Bhyb2_47a

-h128-Bdis (1):Bhyb2_48a

-h129-Bdis (1):Bhyb2_50a

-h130-Bdis (1):Bhyb2_52a

-h131-Bdis (1):Bhyb2_54a

-h132-Bdis (1):Bhyb2_55a

-h133-Bdis (1):Bhyb2_58a

-h134-Bdis (1):Bhyb2_59a

-h135-Bdis (1):Bhyb2_63a

-h136-Bdis (1):Bhyb35_2a

-h137-Bsta (1):Bhyb35_5a

-h138-Bdis (1):Bhyb35_8a

-h139-Bdis (1):Bhyb35_11a

-h140-Bsta (1):Bhyb35_13a

-h141-BdisBsta (1):Bhyb35_15a

-h142-Bdis (1):Bhyb34_1a

-h143-Bdis (1):Bhyb34_2a

-h144-Bsta (1):Bhyb34_5a

-h145-Bsta (1):hyb34_9a

-h146-Bsta (1):Bhyb34_13a

-h147-Bsta (1):Bhyb34_14a

-h148-Bsta (1):Bhyb34_15a

-h149-Bsta (1):Bhyb34_16a

-h150-Bsta (1):Bhyb69_4a

-h151-Bsta (2):Bhyb69_6a, Bhyb69_2a

-h152-Bsta (1):Bhyb69_9a

-h153-Bsta (1):Bhyb69_10a

-h154-Bsta (1):Bhyb69_12a

-h155-Bsta (1):Bhyb69_13a

-h156-Bsta (1):Bhyb69_15a

-h157-Bsta (1):Bhyb69_16a

-h158-Bsta (1):Bhyb69_1a

-h159-Bsta (1):Bhyb69_5a

-h160-Bsta (1):Bhyb69_3a

-h161-Bsta (1):Bhyb69_8a

-h162-Bsta (1):Bhyb69_7a

-h163-Bsta (1):Bhyb69_14a

-h164-Bsta (1):Bhyb69_19a

-h165-Bsta (1):Bhyb69_20a

-h166-Bsta (1):Bhyb69_21a

-h167-Bdis (1):Bhyb69_22a

-h168-Bsta (1):Bhyb69_24a

-h169-Bsta (1):Bhyb69_25a

-h170-Bsta (1):Bhyb69_26a

-h171-Bdis (1):Bhyb69_28a

-h172-Bsta (1):Bhyb69_29a

-h173-Bsta (1):Bhyb69_30a

-h174-Bsta (1):Bhyb69_31a

-h175-Bsta (1):Bhyb69_32a

-h175-Bsta (1):Bhyb69_34a

-h177-Bsta (1):Bhyb69_35a

-h178-Bsta (1):Bhyb69_36a

-h179-Bsta (1):Bhyb69_37a

-h180-Bsta (1):Bhyb69_38a

-h181-Bsta (1):Bhyb69_39a

-h182-Bsta (1):Bhyb69_40a

-h183-Bsta (1):Bhyb69_41a

-h184-Bsta (1):Bhyb69_42a

-h185-Bsta (1):Bhyb69_43a

-h186-Bsta (1):Bhyb69_44a

-h187-Bsta (1):Bhyb69_45a

-h188-Bsta (1):Bhyb69_46a

-h189-Bsta (1):Bhyb69_47a

-h190-Bsta (1):Bhyb69_48a

-h191-Bsta (1):Bhyb69_49a

-h192-Bsta (1):Bhyb69_50a

-h193-Bsta (1):Bhyb69_52a

-h194-Bsta (1):Bhyb69_53a

-h195-Bsta (1):Bhyb69_54a

-h196-Bsta (1):Bhyb69_56a

-h197-Bsta (1):Bhyb69_59a

-h198-Bdis (1):Bhyb69_61a

-h199-Bsta (1):Bhyb69_62a

-h200-Bsta (1):Bhyb69_63a
