# Supplementary material for: A DNA Barcoding Method to Discriminate between the Model Plant Brachypodium distachyon and Its Close Relatives B. stacei and B. hybridum (Poaceae)
Source: PLoS One. 2012 Dec 11;7(12):e51058. doi: 10.1371/journal.pone.0051058 (PMC3519806; doi:10.1371/journal.pone.0051058)
Supplement: Table S3 — Characteristics of the studied Brachypodium distachyon s. l. complex (B. distachyon, B. stacei, B. hybridum) trnLF, ITS and GI sequences. (DOCX) [file pone.0051058.s004.docx]

Table S3. Characteristics of the studied *Brachypodium distachyon* s. l. complex (*B. distachyon, B. stacei, B. hybridum*) *trn*LF, ITS and GI sequences

|  | ***trn*L** | **ITS** | | | | **GI** |
| --- | --- | --- | --- | --- | --- | --- |
|  |  | **ITS1** | **5.8** | **ITS2** | **total** |  |
| Aligned positions | 782 | 222 | 163 | 227 | 612 | 665 |
| Variable positions | 38 (4.9%) | 33 (14.9%) | 19 (11.6%) | 53 (23.3%) | 105 (17.2%) | 263 (39.5%) |
| Potentially informative positions | 25 (3.2%) | 17 (7.6%) | 2 (1.2%) | 24 (10.6%) | 43 (7.0%) | 75 (11.2%) |
| Informative gaps  (positions) | 5  (28-32)  (134-142)  (148)  (445)  (478-483) | 1  (50-51) | - | 2  (402-404)  (490-493) | 3 | 1  (586) |
| Number of haplotypes | 28 | - | - | - | 65 | 200 |
