# Supplementary material for: A DNA Barcoding Method to Discriminate between the Model Plant Brachypodium distachyon and Its Close Relatives B. stacei and B. hybridum (Poaceae)
Source: PLoS One. 2012 Dec 11;7(12):e51058. doi: 10.1371/journal.pone.0051058 (PMC3519806; doi:10.1371/journal.pone.0051058)
Supplement: Methods S1 — Supplemental methods. (DOCX) [file pone.0051058.s005.docx]

Methods S1. Supplemental methods. Amplification conditions of the *trn*LF, ITS and GI loci.

Total DNA was extracted from dried leaf tissue using a modified CTAB method of Doyle and Doyle (1987). The primers used for amplification were ITSL (5’-TCGTAACAAGGTTTCCGTAGGTG-3’) and ITS4 (5’-TCCTCCGCTTATTGATATGC-3’) (Hsiao et al 1995) for the internal transcribed spacer (ITS) of the ribosomal DNA repeat, GIGIE1F (5’-TATGTCWGYNTCAAATGGGAAGTGG-3’) and HGIE5R (5’-AACTTTRAAGATTGGCCTRTTGTRGTGA-3’) for *GIGANTEA* gene (Wolny et al. 2011), and finally the primer ¨c¨ (5’-CGAAATCGGTAGACGCTACG-3’) and ¨f¨(5’-ATTTGAACTGGTGACACGAG-3’) for the intergenic spacer (trnLF) region in the plastid genome (Taberlet et al. 1991). PCR reactions of 25 µl using 1X buffer, 2.5 mM MgCl_2_, 0.2 mM of each dNTP, 0.2 µM of each primer, 0.5 U of Taq polymerase (Roche Applied Science, Germany), and 50ng/ µl of DNA, were performed in an MJ Research PTC-200 thermal cycler (Waltham, Massachusetts) using PCR protocol for ITS: one initial denaturing cycle at 94 °C for 4 min, which was followed by 35 cycles at 94 °C for 45 s, 55 °C for 45 s, 72 °C for 1 min; for GI region: one initial denaturing cycle at 95 °C for 3 min, followed by 30 cycles at 95 °C for 30 s, 58 °C for 30 s, 72 °C for 30 sec, and a final extension step at 72 °C for 7 min; for trnLF region: one initial denaturing cycle at 94 °C for 1 min, followed by 35 cycles at 94 °C for 15 s, 55 °C for 30 s, 72 °C for 1 min, and a final extension step at 72 °C for 7 min. PCR products were purified using ExoSAP-ITTM (USB Corporation, Cleveland, OH) and sequenced in both directions by cycle-sequencing using the Big-Dye version 3 chemistry (Perkin-Elmer), with a Prism 3100 Genetic Analyzer (ABI).
